# Palladium(II)-Catalyzed *othro*-C–H-Benzoxylation of 2-Arylpyridines by Oxidative Coupling with Aryl Acylperoxides

**DOI:** 10.3390/molecules18044403

**Published:** 2013-04-15

**Authors:** Wing-Nga Sit, Chun-Wo Chan, Wing-Yiu Yu

**Affiliations:** State Key Laboratory of Chirosciences and Department of Applied Biology and Chemical Technology, the Hong Kong Polytechnic University, Hung Hom, Kowloon, Hong Kong

**Keywords:** C–H bond, oxidation, palladium

## Abstract

A palladium(II)-catalyzed *ortho*-benzoxylation of 2-arylpyridines with aryl acylperoxides was developed. With pyridyl as directing group, the benzoxylation reaction exhibits remarkable regioselectivity and excellent functional group tolerance, providing the products in up to 87% yield.

## 1. Introduction

Site selective direct functionalization of C–H bonds is attracting considerable current attention for developing sustainable chemical synthesis. Without relying on prefunctionalized substrates, the direct C–H functionalization approach would streamline the synthetic routes and improve atom economy. With transition metal catalysis, innovative transformations based on regioselective aryl C–H bond cleavage with the formation of C–C and C–X (X = halogen) bonds have been achieved [1,2,3]. Recently, significant advances have been made in the analogous transformations with C–O bond formation on arenes. For instance, the research groups of Sanford, Crabtree and Wang achieved acetoxylation of aryl C–H bonds with oxidants such as Oxone^®^, PhI(OAc)_2_, and K_2_S_2_O_8_ [4,5,6]. The analogous acetoxylation of selected sp^3^ C–H bonds has also been achieved independently by Sanford, Yu and Corey [7,8,9]. Notably, Yu and co-workers accomplished Pd-catalyzed arene hydroxylation with dioxygen as oxidant [10]. Apart from Pd, Cu(OAc)_2_ has also been shown to exhibit promising reactivities toward catalytic acetoxylation of aryl C–H bonds [11].

**Figure 1:**
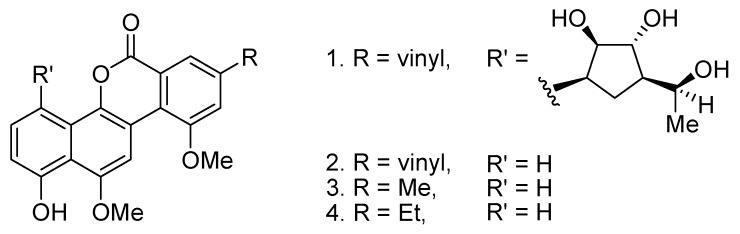
Examples of gilvocarins.

Compared to acetoxylation, the analogous benzoxylation is less developed [12]. Aryl benzoates are known to form structures of some natural products such as the gilvocarcins (Figure 1) [13,14]. In 2009, Cheng and co-workers reported the Rh(I)-catalyzed *ortho*-benzoxylation of 2-arylpyridines with benzoic acids [15]. Later, the same group also reported the analogous Cu(OAc)_2_-catalyzed benzoxylations with benzoic anhydride and derivatives [16,17]. With a continuing interest in developing catalytic C–H bond cross coupling reactions, we have been investigating the regioselective C–C and C–N bond cross coupling reactions based on the coupling reactions of arylpalladium and –rhodium complexes with carboradicals [18,19,20,21], carbenes [22] and nitrenoids [23,24,25]. Previously, we described the successful development of the Pd-catalyzed decarboxylative arylation of 2-arylpyridines with aryl acylperoxides [19]. Prompted by this work, here we disclose the Pd-catalyzed regioselective benzoxylation of 2-arylpyridines with aryl acylperoxides.

## 2. Results and Discussion

**Scheme 1:**
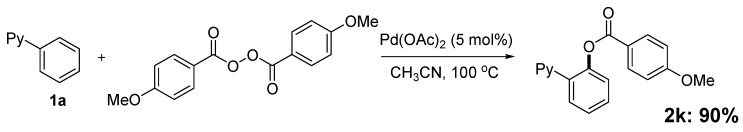
Initial studies of *othro*-C-H-arylcarboxylation by aryl acylperoxide.

In our earlier study on decarboxylative arylation, we found that treating 2-phenylpyridine (**1a**, 0.25 mmol) with Pd(OAc)_2_ (5 mol%) with a methoxy-substituted aryl acylperoxide (4 × 0.5 equiv./0.5 h) at 100 °C for 2 h in CH_3_CN (Scheme 1) furnished **2k** in 90% isolated yield. However, when simple benzoyl peroxide was employed as reagent, the analogous reaction of **1a** produced **2a** in only 7% yield. To develop a general benzoxylation reaction, we first examined the solvent effect (Table 1). With **1a** as substrate and benzoyl peroxide as reagent (4 × 0.5 equiv./0.5 h), the best result (**2a**: 49%) was obtained when xylene was employed as solvent (entry 8). As expected, toluene gave comparable results; the use of other donor solvents (e.g., dioxane, THF, DMF and DCE) produced far inferior outcomes.

We next turned to optimize other experimental parameters, and the results are summarized in Table 2. We found that when raising the reaction temperature to 120 °C led to slightly lower yield of 41% for the benzoxylation of **1a** (entry 2). Notably, further increasing the temperature to 130 °C afforded **2a** in 59% yield (entry 3). Either prolonged reaction time or higher temperature (150 °C) did not give better results (entries 4–5). After several trials, we found that employing 3.5 equiv. of benzoyl peroxide added in a batchwise fashion (7 × 0.5 equiv./0.5 h) improved the benzoxylation yield to 64% (entry 6). Interestingly, when dried benzoyl peroxide was employed, **2a** was obtained in 70% yield. Up to 76% yield of **2a** was obtained when 10 mol% of Pd(OAc)_2_ was employed for the benzoxylation reaction (entry 9).

**Table 1:** Solvent effect. 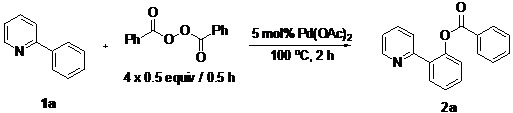

Entry	Solvent (2 mL)	GC Yield (%)
1	CH_3_CN	7
2	dioxane	10
3	THF	29
4	DMF	33
5	DMA	3
6	DCE	11
7	toluene	47
**8**	**xylene**	**49**

**Table 2:** Effect of other experimental parameters. 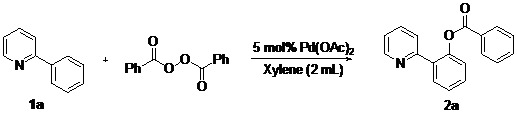

Entry	Addition method	Temp. (°C)	Time (h)	GC Yield (%)
1	4 × 0.5 equiv./0.5 h	100	2	49
2	4 × 0.5 equiv./0.5 h	120	2	41
3	4 × 0.5 equiv./0.5 h	130	2	59
4	4 × 0.5 equiv./1 h	130	4	53
5	4 × 0.5 equiv./0.5 h	150	2	52
6	7 × 0.5 equiv./0.5 h	130	3.5	64
7	8 × 0.5 equiv./0.5 h	130	4	60
**8** *	7 × 0.5 equiv./0.5 h	130	3.5	70
**9** *^,#^	**7 × 0.5 equiv./0.5 h**	**130**	**3.5**	**76**

* Peroxide was washed with diethyl ether and air dry; # 10 mol% of Pd(OAc)_2_ was used.

Table 3 depicts the substrate scope study, and the Pd-catalyzed benzoxylation is broadly applicable to a variety of 2-arylpyridines. In all cases, benzoxylation occurs at the *ortho*-C-H bond to the pyridyl group. For *meta*-substituted arenes (e.g., **1d** and **1e**), benzoxylation occurred regioselectively at the less hindered *ortho*-C-H bond (entries 4–7). Apparently, those aryl groups bearing electron-donating substituent such as Me, MeO produced better yields of **2d** and **2e** (83%–87%), whereas the analogous reactions of substrates with electron-withdrawing CF_3_ and F groups resulted in 54%–62% yields. Higher benzoxylation yield was also observed with **1h**, which contains a Me group on the pyridyl function. Presumably, stronger coordination of the pyridyl group would facilitate cyclopalladation, thereby speeding up the substrate conversion. Consistent with this notion is that benzoxylation of benzoquinoline **1j** was equally facile with **2j** being obtained in 85% yield. The rigid scaffold of **1j** should lower the entropic cost for the cyclopalladation (entry 10). The molecular structure of **2i** has been established by X-ray crystallography. CCDC 933423 contains the supplementary crystallographic data for this paper. These data can be obtained free of charge via www.ccdc.cam.ac.uk/conts/retrieving.html (or from the CCDC, 12 Union Road, Cambridge CB2 1EZ, UK; fax: +44 1223 336033; e-mail: deposit@ccdc.cam.ac.uk), see Supporting Information.

**Table 3:** Pd-catalyzed benzoxylation of arylpyridines *^a^.*

Entry	Substrate	Product		Isolated Yield (%)	
**1**	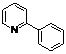 **1a**	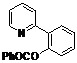 **2a**		71	
**2**	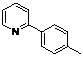 **1b**	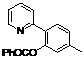 **2b**		70	
**3**	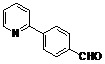 **1c**	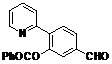 **2c**		44	
**4**	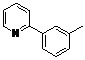 **1d**	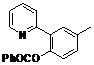 **2d**		87	
**5**	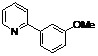 **1e**	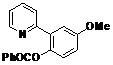 **2e**		83	
**6**	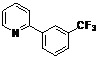 **1f**	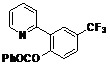 **2f**		54	
**7**	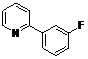 **1g**	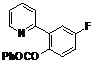 **2g**		62	
**8**	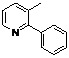 **1h**	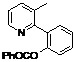 **2h**		87	
**9**	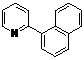 **1i**	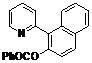 **2i**		57	
**10**	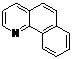 **1j**	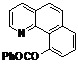 **2j**		85	

*^a^ Reaction conditions*: substrate (0.5 mmol), Pd(OAc)_2_ (5 mol%), peroxide (7 × 0.5 equiv./0.5 h), at 130 °C for 3.5 h in xylene (2 mL).

Table 4 depicts the scope of the aryl acylperoxides; the Pd-catalyzed benzoxylations of **1a** afforded the corresponding benzoates in 27%–81% yields. Peroxides with electron-donating (e.g., Me, MeO, EtO) and -withdrawing (e.g., CF_3_) are effective reagents for the benzoxylation. Peroxides with bulky substituents such as *t*Bu and naphthalene were also tolerated with benzoates being formed in 44%–77% yields.

**Table 4:** Benzoxylation with various aryl acylperoxides *^a^*. 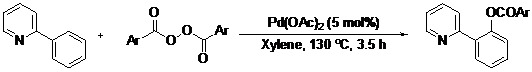

Entry	Ar	Isolated Yield (%)
**1**	4-OMe-C_6_H_5_	54
**2**	3-OMe-C_6_H_5_	69
**3**	2-Cl-C_6_H_5_	43
**4**	3-Cl-C_6_H_5_	73
**5**	2-Me-C_6_H_5_	66
**6**	4-Me-C_6_H_5_	64
**7**	4-F-C_6_H_5_	55
**8**	4-Br-C_6_H_5_	30
**9**	4-CF_3_-C_6_H_5_	81
**10**	4-OEt-C_6_H_5_	27
**11**	4-^t^Bu-C_6_H_5_	77
**12**	2,6-F_2_-C_6_H_4_	46
**13**	2-naphthalene	44

*^a^ Reaction conditions*: substrate (0.5 mmol), Pd(OAc)_2_ (5 mol% ), peroxide (7 × 0.5 equiv./0.5 h), at 130 °C for 3.5 h in xylene (2 mL).

**Scheme 2:**
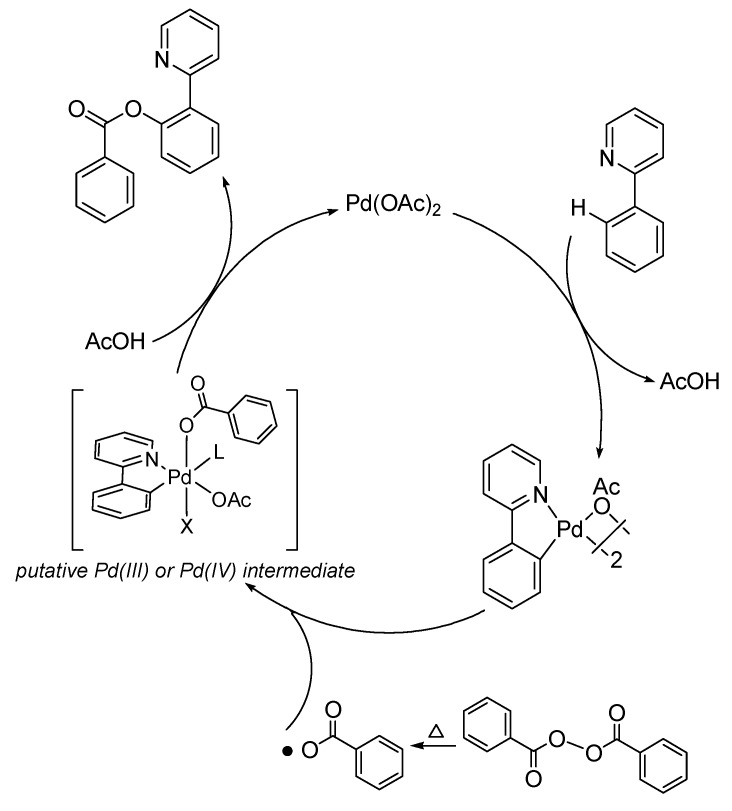
Plausible mechanism of *othro*-C-H-benzoxylation by aryl acylperoxides.

A plausible mechanism is shown in Scheme 2. The benzoxylation is probably initiated by the pyridyl-assisted cyclopalladation of the *ortho*-C-H of the arenes. Our earlier study showed that the regioselectivity of the cyclopalladation is determined by steric factor [19]. Substrates with a *meta*-substituted group would be palladated at the least hindered site. Regarding to the nature of the palladation step, our previous studies established a linear free energy relationship (ρ^+^ = −0.74) for the Pd-catalyzed oxidative acylation of pivilanilides, consistent with an electrophilic mechanism [21]. We conjectured that homolytic O–O cleavage of the aryl acylperoxide should afford arylcarboxy radicals [26], which would react with the palladacyclic intermediate leading to the C–O bond formation.

## 3. Experimental

### 3.1. General

2-Arylpyridines were prepared by reacting the corresponding arylboronic acids with 2-bromopyridines using reported procedures [27]. Aryl acylperoxides were prepared by reacting acid chlorides with hydrogen peroxide (35 wt. % in H_2_O) and sodium hydroxide by the reported procedures [28]. Benzoyl peroxide (Luperox^®^ A75) was obtained commercially and washed with diethyl ether and air dry. Thin layer chromatography was performed on silica gel plates. Silica gel (Merck, 230–400 mesh) and aluminum oxide (Merck, 50–200 mesh) were used for flash column chromatography. ^1^H 400 MHz) and ^13^C-NMR spectra (100 MHz) were recorded on a Brüker DPX 400 NMR spectrometer, chemical shift (δ) valued are given in ppm and are referenced to the residual solvent peaks. Coupling constants (*J*) were reported in Hertz (Hz). Mass spectra and high resolution mass spectra (HRMS) were obtained on a VG Micromass Fison VG platform, a Finnigan Model Mat 95 ST instrument, or a Brüker APEX 47e FT-ICR mass spectrometer. Infrared analysis were measured on a Nicolet Magna 750 FTIR spectrometer. Melting points were measured on a Büchi B-545 melting point apparatus. The X-ray crystal structure was obtained on a Brüker CCD area detector diffractometer.

### 3.2. General Procedure for the Pd-Catalyzed Benzoxylation

A mixture of substrate (0.5 mmol), Pd(OAc)_2_ (0.025 mmol, 5 mol%), aryl acylperoxide [1.75 mmol; addition interval: 7 × 0.5 equiv./0.5 h] in xylene (2 mL) was sealed in a 8-mL vial with a Teflon-lined cap. The mixture was heated at 130 °C (oil bath temperature) for 3.5 h. After cooling down to room temperature, the reaction mixture was filtered through a plug of silica gel, and the filtrate was concentrated under vacuum to afford an oily substance. The crude product was dissolved in dichloromethane and treated with saturated aqueous NaHCO_3_ (3 × 10 mL) solution and extracted with dichloromethane (4 × 10 mL). The combined organic extracts were dried over Na_2_SO_4_ and evaporated to dryness by a rotary evaporator. The residue was loaded to a silica gel column for purification by flash chromatography using 60% *n*-hexane/40% diethyl ether as eluent.

### 3.3. Characterization of Products

*2-(Pyridin-2-yl)phenyl benzoate* (**2a**). Yellow oil (71% yield). ^1^H-NMR (CDCl_3_): *δ_H_* 8.59 (d, *J* = 4.7 Hz, 1H), 8.09 (d, *J* = 7.8 Hz, 2H), 7.78 (d, *J* = 7.6 Hz, 1H), 7.55–7.63 (m, 3H), 7.38–7.52 (m, 4H), 7.31 (d, *J* = 8.0 Hz, 1H), 7.13–7.16 (m, 1H). ^13^C-NMR (CDCl_3_): *δ_C_* 165.2 (C=O), 154.7(C), 150.2 (C-O), 149.4 (C-H), 136.7 (C-H), 134.0 (C-H), 131.5 (C-H), 130.7 (C-H), 130.3 (C), 129.1 (C), 128.9 (C-H), 128.4 (C-H), 127.0 (C-H), 124.2 (C-H), 123.9 (C-H), 122.7 (C-H). MS (EI): 275 (M^+^, 20), 105 (100), 77 (40). HRMS (ESI): calcd. for C_14_H_13_NO_2_H^+^: 276.1025, found: 276.1023. IR (KBr, cm^−1^): 1739.

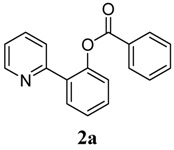


*5-Methyl-2-(pyridin-2-yl)phenyl benzoate* (**2b**). Yellow oil (70% yield). ^1^H-NMR (CDCl_3_): *δ_H_* 8.57 (d, *J* = 4.4 Hz, 1H), 8.10 (d, *J* = 7.5 Hz, 2H), 7.69 (d, *J* = 7.9 Hz, 1H), 7.50–7.61 (m, 3H), 7.45 (t, *J* = 7.7 Hz, 2H), 7.21 (d, *J* = 8.3 Hz, 1H), 6.93–6.97 (m, 1H), 2.43 (s, 3H). ^13^C-NMR (CDCl_3_): *δ_C_* 165.9 (C=O), 156.2 (C), 150.1 (C-O), 149.4 (C-H), 140.8 (C), 136.7 (C-H), 134.0 (C), 131.3 (C-H), 131.0 (C-H), 130.6 (C-H), 130.2 (C), 129.1 (C-H), 128.4 (C-H), 124.2 (C), 122.5 (C-H), 122.1 (C-H), 21.9 (CH_3_). MS (EI): 289 (M^+^, 40), 105 (100), 77 (40). HRMS (ESI): calcd. for C_19_H_15_NO_2_H^+^: 290.1181, found: 290.1188. IR (KBr, cm^−1^): 1736.

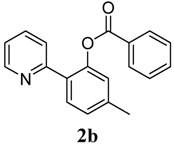


*5-Formyl-2-(pyridin-2-yl)phenyl benzoate* (**2c**). Yellow oil (44% yield based on conversion). ^1^H-NMR (CDCl_3_): *δ_H_* 10.07 (s, 1H), 8.62 (d, *J* = 4.6 Hz, 1H), 8.08 (d, *J* = 7.3 Hz, 2H), 7.95–8.00 (m, 2H), 7.91 (dd, *J* = 7.9, 1.2 Hz, 1H), 7.83 (d, *J* = 1.2 Hz, 1H), 7.60–7.67 (m, 2H), 7.48 (d, *J* = 8.2 Hz, 1H), 7.21–7.23 (m, 1H). ^13^C-NMR (CDCl_3_): *δ_C_* 191.5 (C=O), 165.5 (C=O), 154.9 (C), 150.5 (C-O), 146.7 (C-H), 139.6 (C-H), 137.0 (C-H), 134.5 (C), 132.5 (C-H), 130.9 (C), 129.6 (C-H), 127.4 (C-H), 126.6 (C-H), 125.2 (C-H), 123.6 (C-H), 120.7 (C-H). MS (EI): 303 (M^+^, 10), 105 (100), 77 (30). HRMS (ESI): calcd. for C_19_H_13_NO_3_H^+^: 304.0974, found: 304.0981. IR (KBr, cm^−1^): 1738, 1697.

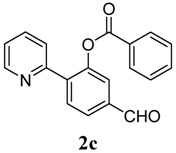


*4-Methyl-2-(pyridin-2-yl)phenyl benzoate* (**2d**). Yellow oil (87% yield). ^1^H-NMR (CDCl_3_): *δ_H_* 8.61 (d, *J* = 4.7 Hz, 1H), 8.09 (d, *J* = 7.4 Hz, 2H), 7.55–7.61 (m, 4H), 7.44 (t, *J* = 7.7 Hz, 2H), 7.28 (dd, *J* = 9.0, 2.1 Hz, 1H), 7.19 (d, *J* = 8.2 Hz, 1H), 7.12–7.15 (m, 1H), 2.44(s, 3H). ^13^C-NMR (CDCl_3_): *δ_C_* 165.9 (C=O), 156.2 (C), 150.2 (C-H), 146.6 (C), 136.7 (C-H), 134.0 (C), 133.4 (C-H), 131.9 (C-H), 130.9 (C), 130.7 (C-H), 130.1 (C-H), 129.1 (C-H), 128.4 (C-H), 124.3 (C-H), 123.6 (C-H), 122.2 (C-H), 21.5 (CH_3_). MS (EI): 289 (M^+^, 30), 105 (100), 77 (30). HRMS (ESI): calcd. for C_19_H_15_NO_2_H^+^: 290.1181, found: 290.1184. IR (KBr, cm^−1^): 1738.

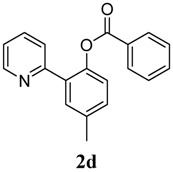


*4-Methoxy-2-(pyridin-2-yl)phenyl benzoate* (**2e**). Yellow oil (83% yield). ^1^H-NMR (CDCl_3_): *δ_H_* 8.61 (d, *J* = 4.7 Hz, 1H), 8.09 (d, *J* = 7.4 Hz, 2H), 7.57–7.62 (m, 2H), 7.45 (t, *J* = 7.7 Hz, 2H), 7.34 (d, *J* = 3.0 Hz, 1H), 7.22 (d, *J* = 8.8 Hz, 1H), 7.13–7.17 (m, 2H), 7.02 (dd, *J* = 8.8, 3.0 Hz, 1H), 3.88 (s, 3H). ^13^C-NMR (CDCl_3_): *δ_C_* 166.1 (C=O), 158.2 (C-O), 156.0 (C), 150.2 (C-H), 149.2 (C-O), 142.4 (C-H), 136.8 (C-H), 134.6 (C-H), 134.0 (C), 131.5 (C-H), 129.1 (C-H), 126.4 (C), 124.8 (C-H), 124.3 (C-H), 116.3 (C-H), 115.8 (C-H), 56.3 (CH_3_). MS (EI): 305 (M^+^, 40), 105 (100), 77 (30). HRMS (ESI): calcd. for C_19_H_15_NO_3_H^+^: 306.1130, found: 306.1133. IR (KBr, cm^−1^): 1737.

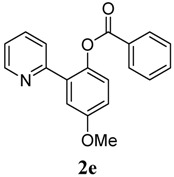


*4-Methoxy-2-(pyridin-2-yl)phenyl benzoate* (**2f**). Yellow oil (54% yield). ^1^H-NMR (CDCl_3_): *δ_H_* 8.63 (d, *J* = 4.5 Hz, 1H), 8.03–8.11 (m, 2H), 7.74 (dd, *J* = 8.5, 1.6 Hz, 1H), 7.57–7.69 (m, 4H), 7.42–7.50 (m, 3H), 7.22 (d, *J* = 5.3 Hz, 1H). ^13^C-NMR (CDCl_3_): *δ_C_* 165.3 (C=O), 154.7 (C), 151.5 (C-O), 150.5 (C-H), 137.1 (C-H), 134.6 (C-H), 134.3 (C-H), 130.9 (C-H), 130.7 (C), 129.5 (C-H), 129.3 (C), 129.1 (C-H), 127.3 (C-H), 124.8 (C-H), 124.4 (CF3), 123.7 (C-H), 123.5 (C-H). MS (EI): 343 (M^+^, 20), 105 (100), 77 (40). HRMS (ESI): calcd. for C_19_H_12_NO_2_F_3_H^+^: 344.0898 found: 344.0905. IR (KBr, cm^−1^): 1739.

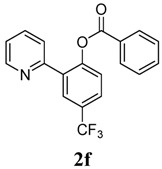


*4-Fluoro-2-(pyridin-2-yl)phenyl benzoate* (**2g**). Yellow oil (62% yield). ^1^H-NMR (CDCl_3_): *δ_H_* 8.60 (d, *J* = 4.7 Hz, 1H), (d, *J* = 4.7 Hz, 1H), 8.08 (d, *J* = 7.4 Hz, 2H), 7.52–7.64 (m, 4H), 7.46 (t, *J* = 7.7 Hz, 2H), 7.25–7.28 (m, 1H), 7.13–7.18 (m, 2H). ^13^C-NMR (CDCl_3_): *δ_C_* 165.8 (C=O), 159.8 (C-F), 154.9 (C), 150.4 (C-H), 144.7 (C-O), 136.9 (C-H), 135.4 (C-H), 134.2 (C-H), 130.8 (C-H), 129.8 (C), 129.2 (C-H), 125.5 (C), 124.2 (C-H), 123.2 (C-H), 117.9 (C-H), 117.1 (C-H). MS (EI): 293 (M^+^, 20), 105 (100), 77 (40). HRMS (ESI): calcd. for C_18_H_12_NO_2_FH^+^: 294.0930, found: 294.0927. IR (KBr, cm^−1^): 1741.

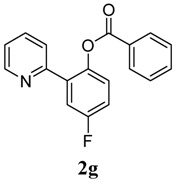


*2-(3-Methylpyridin-2-yl)phenyl benzoate* (**2h**). Yellow oil (87% yield). ^1^H-NMR (CDCl_3_): *δ_H_* 8.42 (d, *J* = 4.5 Hz, 1H), 7.89 (d, *J* = 7.5 Hz, 1H), 7.26–7.54 (m, 8H), 7.04–7.07 (m, 1H), 2.21 (s, 3H). ^13^C-NMR (CDCl_3_): *δ_C_* 165.0 (C=O), 155.9 (C), 148.8 (C-O), 147.1 (C-H), 138.3 (C-H), 134.0 (C-H), 133.8 (C-H), 132.6 (C), 130.9 (C-H), 130.6 (C), 130.1 (C-H), 129.7 (C), 128.8 (C-H), 126.4 (C-H), 123.3 (C-H), 123.0 (C-H), 19.5 (CH_3_). MS (EI): 289 (M^+^, 30), 105 (100), 77 (40). HRMS (ESI): calcd. for C_19_H_15_NO_2_H^+^: 290.1181, found: 290.1176. IR (KBr, cm^−1^): 1736.

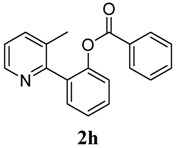


*1-(Pyridin-2-yl)naphthalen-2-yl benzoate* (**2i**). Yellow oil (57% yield). ^1^H-NMR (CDCl_3_): *δ_H_* 8.77 (d, *J* = 4.2 Hz, 1H), 7.93–8.01 (m, 4H), 7.71 (t, *J* = 7.6 Hz, 1H), 7.63 (d, *J* = 8.2 Hz, 1H), 7.55–7.57 (m, 1H), 7.44–7.54 (m, 4H), 7.40 (t, *J* = 7.6 Hz, 2H), 7.24–7.27 (m, 1H). ^13^C-NMR (CDCl_3_): *δ_C_* 165.9 (C=O), 155.4 (C), 150.3 (C-O), 146.7 (C-H), 136.7 (C-H), 134.0 (C-H), 133.4 (C), 132.5 (C-H), 130.6 (C-H), 130.1 (C), 129.8 (C), 129.0 (C-H), 128.8 (C-H), 128.7 (C), 127.7 (C-H), 127.5 (C-H), 126.4 (C-H), 126.2 (C-H), 123.0 (C-H), 122.3 (C-H). MS (EI): 325 (M^+^, 50), 191 (10) 105 (100), 77 (40). HRMS (ESI): calcd. for C_22_H_15_NO_2_H^+^: 326.1130, found: 326.1129. IR (KBr, cm^−1^): 1735.

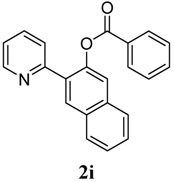


*Benzo[h]quinolin-10-yl benzoate* (**2j**). Yellow oil (85% yield). ^1^H-NMR (CDCl_3_): *δ_H_* 8.39–8.42 (m, 3H), 8.08 (dd, *J* = 8.0, 1.8 Hz, 1H), 7.83–7.89 (m, 2H), 7.66–7.75 (m, 3H), 7.53–7.62 (m, 3H), 7.33 (q, *J* = 4.1 Hz, 1H). ^13^C-NMR (CDCl_3_): *δ_C_* 167.5 (C=O), 149.7 (C-O), 148.5 (C-H), 146.1 (C), 136.6 (C-H), 136.1 (C), 133.3 (C-H), 132.0 (C), 131.1 (C-H), 129.0 (C-H), 128.7 (C), 128.5 (C-H), 127.6 (C-H), 127.3 (C-H), 127.0 (C-H), 124.0 (C), 123.0 (C-H), 122.1 (C-H). MS (EI): 299 (M^+^, 40), 105 (100), 77 (30). HRMS (ESI): calcd. for C_20_H_13_NO_2_H^+^: 300.1025, found: 300.1024. IR (KBr, cm^−1^): 1735.

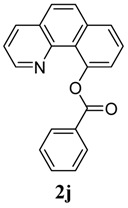


*2-(Pyridin-2-yl)phenyl 4-methoxybenzoate* (**2k**). Yellow oil (54% yield). ^1^H-NMR (CDCl_3_): *δ_H_* 8.59 (d, *J* = 4.5 Hz, 1H), 8.03 (d, *J* = 8.8 Hz, 2H), 7.78 (dd, *J* = 7.6, 1.5 Hz, 1H), 7.54–7.63 (m, 2H), 7.45 (dt, *J* = 7.6, 1.5 Hz, 1H), 7.36 (dt, *J* = 7.5, 0.8 Hz, 1H), 7.29 (dd, *J* = 7.9, 0.7 Hz, 1H), 7.11–7.14 (m, 1H), 6.91 (d, *J* = 8.8 Hz, 2H), 3.82 (s, 3H). ^13^C-NMR (CDCl_3_): *δ_C_* 165.4 (C=O), 164.3 (C), 156.1 (C), 150.1 (C), 148.9 (C-H), 136.6 (C-H), 133.9 (C-H), 132.8 (C-H), 131.4 (C-H), 130.2 (C), 126.8 (C-H), 123.9 (C-H), 122.6 (C), 122.2 (C-H), 119.2 (C-H), 114.3 (C-H), 56.0 (CH_3_). MS (EI): 305 (M^+^, 20), 135 (100). HRMS (ESI): calcd. for C_19_H_15_NO_3_H^+^: 306.1130, found: 306.1136. IR (KBr, cm^−1^): 1732.

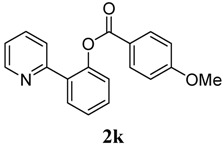


*2-(Pyridin-2-yl)phenyl 3-methoxybenzoate* (**2l**). Yellow oil (69% yield). ^1^H-NMR (CDCl_3_): *δ_H_* 8.60 (d, *J* = 4.1 Hz, 1H), 7.78 (dd *J* = 7.7, 1.5 Hz, 1H), 7.69 (d, *J* = 6.6 Hz, 1H), 7.55–7.64 (m, 3H), 7.47 (dt *J* = 7.7, 1.6 Hz, 1H), 7.35–7.41 (m, 2H), 7.32 (dt *J* = 8.0, 1.0 Hz, 1H), 7.10–7.17 (m, 2H), 3.81 (s, 3H). ^13^C-NMR (CDCl_3_): *δ_C_* 165.6 (C=O), 160.2 (C), 156.1 (C), 150.2 (C), 148.9 (C-H), 136.8 (C-H), 133.9 (C-H), 131.5 (C), 131.3 (C-H), 130.3 (C-H), 130.1 (C), 127.0 (C-H), 124.3 (C-H), 123.9 (C-H), 123.2 (C-H), 122.8 (C-H), 120.7 (C-H), 115.0 (C-H), 56.0 (CH_3_). MS (EI): 305 (M^+^, 20), 135 (100). HRMS (ESI): calcd. for C_19_H_15_NO_3_H^+^: 306.1130, found: 306.1134. IR (KBr, cm^−1^): 1736.

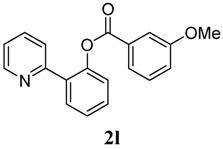


*2-(Pyridin-2-yl)phenyl 2-chlorobenzoate* (**2m**). Yellow oil (43% yield). ^1^H-NMR (CDCl_3_): *δ_H_* 8.63 (d, *J* = 4.7 Hz, 1H), 7.88 (dd, *J* = 7.8, 1.2 Hz, 1H), 7.74 (dd, *J* = 7.7, 1.5 Hz, 1H), 7.68 (dt, *J* = 7.7, 1.7 Hz, 1H), 7.57 (d, *J* = 7.8 Hz, 1H), 7.37–7.52 (m, 4H), 7.31–7.34 (m, 2H), 7.18–7.21 (m, 1H). ^13^C-NMR (CDCl_3_): *δ_C_* 165.2 (C=O), 156.4 (C), 150.2 (C-O), 148.7 (C-H), 137.0 (C-H), 135.0 (C-Cl), 134.0 (C-H), 133.6 (C-H), 132.5 (C-H), 131.8 (C-H), 131.5 (C), 130.5 (C-H), 130.0 (C), 127.2 (C-H), 124.4 (C-H), 124.0 (C-H), 122.9 (C-H), 122.1 (C-H). MS (EI): 309 (M^+^, 40), 139 (100), 111 (20). HRMS (ESI): calcd. for C_18_H_12_NO_2_ClH^+^: 310.0635, found: 310.0628. IR (KBr, cm^−1^): 1742.

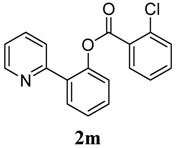


*2-(Pyridin-2-yl)phenyl 3-chlorobenzoate* (**2n**). Yellow oil (73% yield). ^1^H-NMR (CDCl_3_): *δ_H_* 8.57 (d, *J* = 4.6 Hz, 1H), 8.06 (s, 1H), 7.95 (d, *J* = 7.8 Hz, 1H), 7.75 (dd, *J* = 7.7, 1.3 Hz, 1H), 7.65 (t, *J* = 7.8 Hz, 1H), 7.53–7.57 (m, 2H), 7.48 (t, *J* = 7.7 Hz, 1H), 7.37–7.42 (m, 2H), 7.30 (d, *J* = 4.5 Hz, 1H), 7.14–7.18 (m, 1H). ^13^C-NMR (CDCl_3_): *δ_C_* 164.6 (C=O), 156.1 (C), 150.2 (C-O), 148.7 (C-H), 136.9 (C-H), 135.2 (C-Cl), 134.0 (C-H), 133.8 (C), 131.9 (C-H), 131.5 (C-H), 130.7 (C-H), 130.4 (C-H), 129.9 (C), 128.8 (C-H), 127.2 (C-H), 124.2 (C-H), 123.8 (C-H), 122.8 (C-H). MS (EI): 309 (M^+^, 20), 139 (100), 111 (40). HRMS (ESI): calcd. for C_18_H_12_NO_2_ClH^+^: 310.0635, found: 310.0640. IR (KBr, cm^−1^): 1740.

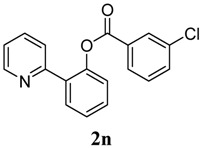


*2-(Pyridin-2-yl)phenyl 2-methylbenzoate* (**2o**). Yellow oil (66% yield). ^1^H-NMR (CDCl_3_): *δ_H_* 8.61 (dd, *J* = 4.8, 0.8 Hz, 1H), 8.01 (dd, *J* = 7.1, 1.3 Hz, 1H), 7.76 (dd, *J* = 7.6, 1.6 Hz, 1H), 7.64 (dt, *J* = 7.7, 1.7 Hz, 1H), 7.56 (d, *J* = 7.9 Hz, 1H), 7.49 (dt, *J* = 7.7, 1.6 Hz, 1H), 7.38–7.45 (m, 2H), 7.24–7.30 (m, 4H), 7.15–7.19 (m, 1H), 2.54 (s, 3H). ^13^C-NMR (CDCl_3_): *δ_C_* 166.3 (C=O), 156.5 (C), 150.2 (C-O), 150.0 (C-H), 141.8 (C), 136.8 (C-H), 134.2 (C-H), 133.1 (C-H), 132.7 (C-H), 132.4 (C-H), 131.5 (C-H), 130.4 (C), 129.2 (C), 128.5 (C-H), 126.9 (C-H), 126.4 (C-H), 124.1 (C-H), 122.8 (C-H), 22.2 (CH_3_). MS (EI): 289 (M^+^, 10), 119 (100), 91 (40). HRMS (ESI): calcd. for C_19_H_15_NO_2_H^+^: 290.1181, found: 290.1186. IR (KBr, cm^−1^): 1738.

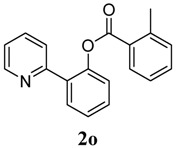


*2-(Pyridin-2-yl)phenyl 4-methylbenzoate* (**2p**). Yellow oil (64% yield). ^1^H-NMR (CDCl_3_): *δ_H_* 8.60 (d, *J* = 4.7 Hz, 1H), 7.98 (d, *J* = 8.1 Hz, 2H), 7.79 (dd, *J* = 7.7, 1.5 Hz, 1H), 7.55–7.60 (m, 2H), 7.47 (dt, *J* = 7.7, 1.5 Hz, 1H), 7.39 (t, *J* = 7.5 Hz, 1H), 7.25 (d, *J* = 7.9 Hz, 2H), 7.14–7.16 (m, 1H), 7.04 (t, *J* = 4.7 Hz, 1H), 2.41 (s, 3H). ^13^C-NMR (CDCl_3_): *δ_C_* 165.8 (C=O), 156.2 (C), 150.2 (C-O), 149.4 (C-H), 144.9 (C), 136.7 (C-H), 134.0 (C-H), 131.5 (C-H), 130.7 (C-H), 130.1 (C-H), 129.6 (C), 127.3 (C), 126.9 (C-H), 124.4 (C-H), 124.0 (C-H), 122.7 (C-H), 22.3 (CH_3_). MS (EI): 289 (M^+^, 10), 119 (100), 91 (40). HRMS (ESI): calcd. for C_19_H_15_NO_2_H^+^: 290.1181, found: 290.1173. IR (KBr, cm^−1^): 1737.

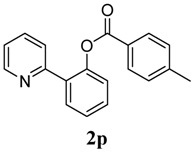


*2-(Pyridin-2-yl)phenyl 4-fluorobenzoate* (**2q**). Yellow oil (55% yield). ^1^H-NMR (CDCl_3_): *δ_H_* 8.57 (d, *J* = 4.2 Hz, 1H), 8.08–8.11 (m, 2H), 7.76 (dd, *J* = 7.6, 1.5 Hz, 1H), 7.62 (dt, *J* = 7.7, 1.6 Hz, 1H), 7.53 (d, *J* = 7.9 Hz, 1H), 7.47 (dt, *J* = 7.7, 1.5 Hz, 1H), 7.39 (dt, *J* = 7.5, 0.9 Hz, 1H), 7.30 (dd, *J* = 7.9, 0.8 Hz, 1H), 7.09–7.16 (m, 3H). ^13^C-NMR (CDCl_3_): *δ_C_* 167.9 (C-F), 164.8 (C=O), 156.1 (C), 150.1 (C-O), 148.8 (C-H), 136.8 (C-H), 133.9 (C-H), 133.4 (C-H), 131.5 (C-H), 130.3 (C), 127.1 (C-H), 126.3 (C), 123.0 (C-H), 122.8 (C-H), 116.4 (C-H), 116.2 (C-H). MS (EI): 293 (M^+^, 10), 123 (100), 95 (30). HRMS (ESI): calcd. for C_18_H_12_NO_2_FH^+^: 294.0930, found: 294.0931. IR (KBr, cm^−1^): 1738.

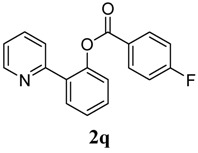


*2-(Pyridin-2-yl)phenyl 4-bromobenzoate* (**2r**). Yellow oil (30% yield). ^1^H-NMR (CDCl_3_): *δ_H_* 8.56 (d, *J* = 4.3 Hz, 1H), 7.94 (d, *J* = 8.5 Hz, 2H), 7.76 (dd, *J* = 7.6, 1.4 Hz, 1H), 7.64 (dd, *J* = 7.8, 1.6 Hz, 1H), 7.46–7.53 (m, 3H), 7.40 (t, *J* = 7.0 Hz, 1H), 7.30 (d, *J* = 7.8 Hz, 1H), 7.14–7.17 (m, 1H). ^13^C-NMR (CDCl_3_): *δ_C_* 165.1 (C=O), 156.2 (C), 150.2 (C-O), 148.8 (C-H), 136.9 (C-H), 133.8 (C-H), 132.5 (C-H), 132.3 (C-H), 131.9 (C-H), 130.4 (C-H), 129.3 (C), 129.1 (C), 127.2 (C-Br), 124.2 (C-H), 123.9 (C-H), 122.8 (C-H). MS (EI): 355 (M^+^, 20), 183 (100), 155 (20). HRMS (ESI): calcd. for C_18_H_12_NO_2_BrH^+^: 354.0130, found: 354.0133. IR (KBr, cm^−1^): 1737.

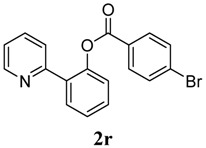


*2-(Pyridin-2-yl)phenyl 4-(trifluoromethyl)benzoate* (**2s**). Yellow oil (81% yield). ^1^H-NMR (CDCl_3_): *δ_H_* 8.54 (d, *J* = 4.5 Hz, 1H), 8.19 (d, *J* = 8.4 Hz, 2H), 7.71–7.82 (m, 3H), 7.64 (dt, *J* = 7.8, 1.7 Hz, 1H), 7.47–7.54 (m, 2H), 7.41 (dt, *J* = 7.5, 1.0 Hz, 1H), 7.31 (d, *J* = 8.0 Hz, 1H), 7.14–7.17 (m, 1H). ^13^C-NMR (CDCl_3_): *δ_C_* 164.7 (C=O), 156.2 (C), 150.1 (C-O), 148.7 (C-H), 137.0 (C), 135.6 (C-H), 135.2 (C-H), 133.8 (C-H), 133.4 (C), 131.5 (C-H), 131.2 (C-H), 130.5 (C), 127.3 (C-H), 125.6 (C-H), 124.1 (CF_3_), 123.9 (C-H), 122.9 (C-H). MS (EI): 343 (M^+^, 40), 173 (100), 145 (50). HRMS (ESI): calcd. for C_19_H_12_NO_2_F_3_H^+^: 344.0898, found: 344.0901. IR (KBr, cm^−1^): 1743.

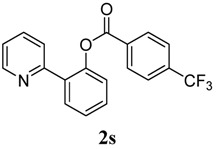


*2-(Pyridin-2-yl)phenyl 4-ethoxybenzoate* (**2t**). Yellow oil (27% yield). ^1^H-NMR (CDCl_3_): *δ_H_* 8.61 (d, *J* = 4.6 Hz, 1H), 8.02 (d, *J* = 8.8 Hz, 2H), 7.78 (dd, *J* = 7.6, 1.3 Hz, 1H), 7.54–7.63 (m, 2H), 7.47 (dt, *J* = 7.6, 1.3 Hz, 1H), 7.38 (dt, *J* = 7.5, 0.9 Hz, 1H), 7.29 (d, *J* = 8.1 Hz, 1H), 7.13–7.17 (m, 1H), 6.91 (d, *J* = 8.8 Hz, 2H), 4.09 (q, *J* = 7.0 Hz, 2H), 1.44 (t, *J* = 7.0 Hz, 1H). ^13^C-NMR (CDCl_3_): *δ_C_* 165.5 (C=O), 163.9 (C-O), 156.2 (C), 150.3 (C-O), 149.1 (C-H), 136.7 (C-H), 134.0 (C-H), 133.0 (C-H), 131.5 (C-H), 130.3 (C), 126.9 (C-H), 124.4 (C-H), 124.1 (C-H), 122.7 (C-H), 122.2 (C), 114.9 (C-H), 64.4 (CH_2_), 15.3 (CH_3_). MS (EI): 319 (M^+^, 10), 207 (20), 149 (100), 121 (40). HRMS (ESI): calcd. for C_20_H_17_NO_3_H^+^: 320.1287, found: 320.1285. IR (KBr, cm^−1^): 1731.

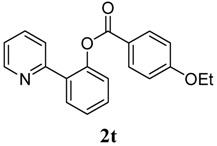


*2-(Pyridin-2-yl)phenyl 4-tert-butylbenzoate* (**2u**). Yellow oil (77% yield). ^1^H-NMR (CDCl_3_): *δ_H_* 8.62 (d, *J* = 4.6 Hz, 1H), 8.04 (d, *J* = 8.3 Hz, 2H), 7.88 (d, *J* = 8.3 Hz, 2H), 7.45–7.54 (m, 2H), 7.36–7.42 (m, 2H), 7.11–7.14 (m, 1H), 1.33 (d, *J* = 13.6 Hz, 9H). ^13^C-NMR (CDCl_3_): *δ_C_* 165.2 (C=O),157.7 (C), 156.1 (C-O), 149.9 (C), 148.9 (C-H), 136.7 (C-H), 133.9 (C-H), 131.5 (C-H), 130.5 (C-H), 130.2 (C), 126.8 (C), 125.9 (C-H), 124.3 (C-H), 123.9 (C-H), 122.6 (C-H), 121.2 (C-H), 35.6 (C), 31.6 (CH_3_). MS (EI): 331 (M^+^, 10), 161 (100), 146 (10). HRMS (ESI): calcd. for C_22_H_21_NO_2_H^+^: 332.1651, found: 332.1652. IR (KBr, cm^−1^): 1737cm^−1^.

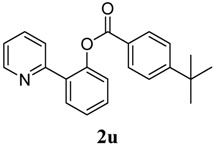


*2-(Pyridin-2-yl)phenyl 2,6-difluorobenzoate* (**2v**). Yellow oil (46% yield). ^1^H-NMR (CDCl_3_): *δ_H_* 8.65 (d, *J* = 4.4 Hz, 1H), 7.76 (dd, *J* = 7.6,1.6 Hz, 1H), 7.70 (dt, *J* = 7.7, 1.7 Hz, 1H), 7.59 (d, *J* = 7.9 Hz, 1H), 7.49 (dt, *J* = 7.7, 1.6 Hz, 1H), 7.39–7.44 (m, 2H), 7.33 (dd, *J* = 8.0, 1.0 Hz, 1H), 7.20–7.23 (m, 1H), 6.95 (t, *J* = 8.3 Hz, 2H). ^13^C-NMR (CDCl_3_): *δ_C_* 162.7 (C=O), 160.7 (C-F), 156.0 (C), 150.2 (C-O), 148.3 (C-H), 136.9 (C-H), 134.1 (C-H), 134.0 (C), 131.6 (C-H), 130.5 (C-H), 127.5 (C-H), 124.4 (C-H), 123.7 (C-H), 122.9 (C-H), 112.9 (C), 112.6 (C-H). MS (EI): 311 (M^+^, 40), 141 (100), 113 (10). HRMS (ESI): calcd. for C_18_H_11_NO_2_F_2_H^+^: 312.0836, found: 312.0841. IR (KBr, cm^−1^): 1751.

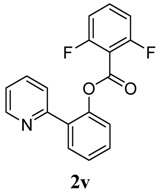


*2-(Pyridin-2-yl)phenyl 2-naphthoate* (**2w**). Yellow oil (44% yield). ^1^H-NMR (CDCl_3_): *δ_H_* 8.69 (s, 1H), 8.59 (d, *J* = 4.8 Hz, 1H), 8.10 (dd, *J* = 8.6, 1.4 Hz, 1H), 7.95 (d, *J* = 8.0 Hz, 1H), 7.90 (d, *J* = 8.5 Hz, 2H), 7.82 (dd, *J* = 7.6, 1.5 Hz, 1H), 7.56–7.63 (m, 4H), 7.51 (dt, *J* = 7.8, 1.6 Hz, 1H), 7.41–7.45 (m, 1H), 7.38 (dd, *J* = 7.9, 0.7 Hz, 1H),7.11–7.15 (m, 1H). ^13^C-NMR (CDCl_3_): *δ_C_* 166.0 (C=O), 156.3 (C), 150.3 (C-O), 149.1 (C-H), 136.8 (C-H), 136.4 (C), 134.0 (C-H), 133.1 (C), 132.6 (C-H), 131.6 (C-H), 130.4 (C-H), 130.1 (C-H), 129.2 (C), 128.9 (C-H), 128.4 (C), 127.4 (C-H), 127.3 (C-H), 127.1 (C-H), 126.1 (C-H), 124.3 (C-H), 124.0 (C-H), 122.8 (C-H). MS (EI): 325 (M^+^, 20), 155 (100), 127 (60). HRMS (ESI): calcd. for C_22_H_15_NO_2_H^+^: 326.1181, found: 326.1191. IR (KBr, cm^−1^): 1734 cm^−1^.

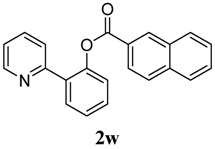


## 4. Conclusions

In summary, we developed a Pd-catalyzed regioselective C–H benzoxylation reaction with aryl acylperoxides as reagents. This catalytic protocol was convenient to operate, and the product benzoates were formed in good yield with high regioselectivity and functional group tolerance.

## Supplementary Materials

Supplementary materials can be accessed at: http://www.mdpi.com/1420-3049/18/4/4403/s1.
