# Supplementary material for: Palladium(II)-Catalyzed othro-C–H-Benzoxylation of 2-Arylpyridines by Oxidative Coupling with Aryl Acylperoxides
Source: Molecules. 2013 Apr 15;18(4):4403–18. doi: 10.3390/molecules18044403 (PMC6270440; doi:10.3390/molecules18044403)

Supplementary Materials

Table of Contents

1. X-ray crystallographic data for 2i **S1**
2. ^{1}H and ^{13}C-NMR spectra **S7**

1. X-ray crystallographic data for 2i:

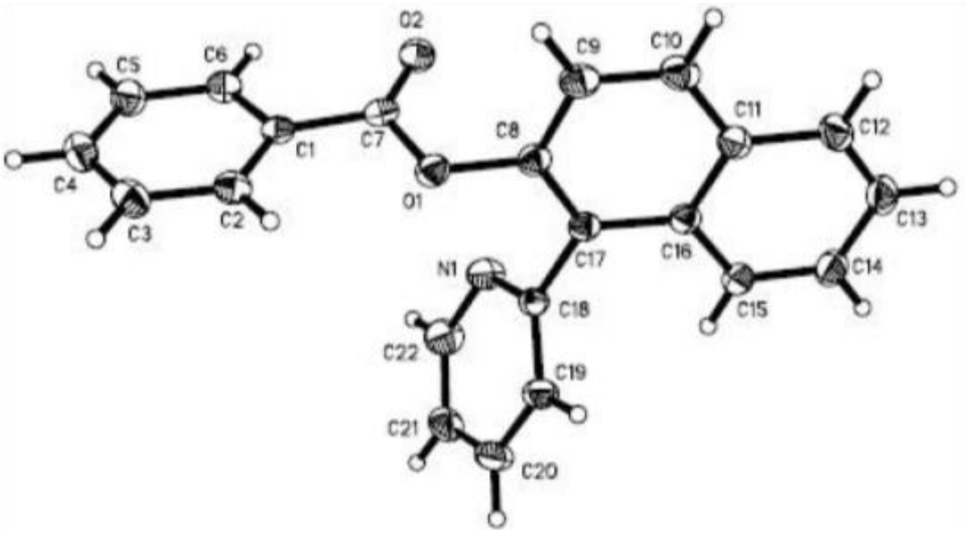

Table S1. Crystal data and structure refinement for **2i** (15 January 2009).

Identification code	snow20	
Empirical formula	$C_{22}H_{15}NO_2$	
Formula weight	325.35	
Temperature	296(2) K	
Wavelength	0.71073 Å	
Crystal system	Triclinic	
Space group	P-1	
Unit cell dimensions	a = 9.0621(2) Å	α = 87.277(2) °.
	b = 9.8863(2) Å	β = 67.4190(10) °.
	c = 10.2723(2) Å	γ = 79.943(2)°.
Volume	836.50(3) Å^3	
Z	2	
Density (calculated)	1.292 Mg/m^3	
Absorption coefficient	0.083 mm^{-1}	
F(000)	340	
Crystal size	0.50 × 0.40 × 0.28 mm^3	
Theta range for data collection	2.09 to 27.68 °.	
Index ranges	$-11 \le h \le 11$, $-12 \le k \le 12$, $-13 \le l \le 13$	
Reflections collected	17175	
Independent reflections	3858 [R(int) = 0.0446]	
Completeness to theta = 27.68 °	98.9%	
Absorption correction	Semi-empirical from equivalents	
Max. and min. transmission	1.000 and 0.817	
Refinement method	Full-matrix least-squares on F^2	
Data/restraints/parameters	3858/0/226	
Goodness-of-fit on F^2	1.001	
Final R indices [I>2sigma(I)]	R1 = 0.0446, wR2 = 0.1112	
R indices (all data)	R1 = 0.1025, wR2 = 0.1364	
Largest diff. peak and hole	0.120 and −0.151 e.Å^{-3}	

Table S2. Atomic coordinates ($\times 10^4$) and equivalent isotropic displacement parameters ($Å^2 \times 10^3$) for **2i**. U(eq) is defined as one third of the trace of the orthogonalized U^{ij} tensor.

	**x**	**y**	**z**	**U(eq)**
O(1)	7689(1)	6326(1)	4917(1)	67(1)
O(2)	5083(1)	7352(1)	5617(1)	79(1)
N(1)	7646(1)	8373(1)	7096(2)	82(1)
C(1)	6982(2)	7965(1)	3431(1)	56(1)
C(2)	8495(2)	7609(1)	2366(2)	69(1)
C(3)	8888(2)	8318(2)	1117(2)	82(1)
C(4)	7799(2)	9380(2)	942(2)	85(1)
C(5)	6303(2)	9754(2)	1990(2)	81(1)
C(6)	5888(2)	9046(1)	3234(2)	68(1)
C(7)	6452(2)	7219(1)	4763(1)	60(1)
C(8)	7314(2)	5561(1)	6168(2)	62(1)
C(9)	6573(2)	4411(1)	6246(2)	74(1)
C(10)	6297(2)	3619(1)	7402(2)	77(1)
C(11)	6737(2)	3926(1)	8513(2)	66(1)
C(12)	6389(2)	3141(1)	9748(2)	81(1)
C(13)	6772(2)	3473(2)	10827(2)	86(1)
C(14)	7535(2)	4611(2)	10727(2)	82(1)
C(15)	7883(2)	5399(1)	9554(2)	69(1)
C(16)	7493(2)	5089(1)	8414(1)	58(1)
C(17)	7786(1)	5919(1)	7195(1)	56(1)
C(18)	8578(2)	7160(1)	7038(1)	56(1)
C(19)	10179(2)	7034(2)	6840(2)	75(1)
C(20)	10869(2)	8189(2)	6681(2)	91(1)
C(21)	9937(2)	9436(2)	6735(2)	90(1)
C(22)	8366(2)	9480(2)	6936(2)	99(1)

Table S3. Bond lengths [Å] and angles [°] for **2i**.

O(1)-C(7)	1.3563(16)
O(1)-C(8)	1.4143(16)
O(2)-C(7)	1.2018(15)
N(1)-C(18)	1.3301(16)
N(1)-C(22)	1.3419(19)
C(1)-C(2)	1.3846(17)
C(1)-C(6)	1.3885(18)
C(1)-C(7)	1.4736(18)
C(2)-C(3)	1.384(2)
C(2)-H(2A)	0.9300
C(3)-C(4)	1.366(2)
C(3)-H(3A)	0.9300
C(4)-C(5)	1.370(2)
C(4)-H(4A)	0.9300
C(5)-C(6)	1.379(2)
C(5)-H(5A)	0.9300
C(6)-H(6A)	0.9300

Table S3. *Cont.*

C(8)-C(17)	1.362(2)
C(8)-C(9)	1.4029(19)
C(9)-C(10)	1.356(2)
C(9)-H(9A)	0.9300
C(10)-C(11)	1.407(2)
C(10)-H(10A)	0.9300
C(11)-C(12)	1.413(2)
C(11)-C(16)	1.4188(18)
C(12)-C(13)	1.351(2)
C(12)-H(12A)	0.9300
C(13)-C(14)	1.401(2)
C(13)-H(13A)	0.9300
C(14)-C(15)	1.365(2)
C(14)-H(14A)	0.9300
C(15)-C(16)	1.406(2)
C(15)-H(15A)	0.9300
C(16)-C(17)	1.4269(18)
C(17)-C(18)	1.4981(17)
C(18)-C(19)	1.3693(18)
C(19)-C(20)	1.370(2)
C(19)-H(19A)	0.9300
C(20)-C(21)	1.358(2)
C(20)-H(20A)	0.9300
C(21)-C(22)	1.351(2)
C(21)-H(21A)	0.9300
C(22)-H(22A)	0.9300
C(7)-O(1)-C(8)	116.89(10)
C(18)-N(1)-C(22)	116.23(13)
C(2)-C(1)-C(6)	119.28(13)
C(2)-C(1)-C(7)	122.78(12)
C(6)-C(1)-C(7)	117.91(11)
C(3)-C(2)-C(1)	119.72(13)
C(3)-C(2)-H(2A)	120.1
C(1)-C(2)-H(2A)	120.1
C(4)-C(3)-C(2)	120.24(14)
C(4)-C(3)-H(3A)	119.9
C(2)-C(3)-H(3A)	119.9
C(3)-C(4)-C(5)	120.73(15)
C(3)-C(4)-H(4A)	119.6
C(5)-C(4)-H(4A)	119.6
C(4)-C(5)-C(6)	119.65(15)
C(4)-C(5)-H(5A)	120.2
C(6)-C(5)-H(5A)	120.2
C(5)-C(6)-C(1)	120.37(13)
C(5)-C(6)-H(6A)	119.8
C(1)-C(6)-H(6A)	119.8
O(2)-C(7)-O(1)	122.85(13)
O(2)-C(7)-C(1)	124.89(13)
O(1)-C(7)-C(1)	112.25(11)

Table S3. *Cont.*

C(17)-C(8)-C(9)	123.21(13)
C(17)-C(8)-O(1)	118.59(11)
C(9)-C(8)-O(1)	118.04(13)
C(10)-C(9)-C(8)	118.72(15)
C(10)-C(9)-H(9A)	120.6
C(8)-C(9)-H(9A)	120.6
C(9)-C(10)-C(11)	121.51(13)
C(9)-C(10)-H(10A)	119.2
C(11)-C(10)-H(10A)	119.2
C(10)-C(11)-C(12)	121.82(13)
C(10)-C(11)-C(16)	119.05(13)
C(12)-C(11)-C(16)	119.08(15)
C(13)-C(12)-C(11)	121.24(15)
C(13)-C(12)-H(12A)	119.4
C(11)-C(12)-H(12A)	119.4
C(12)-C(13)-C(14)	119.87(15)
C(12)-C(13)-H(13A)	120.1
C(14)-C(13)-H(13A)	120.1
C(15)-C(14)-C(13)	120.57(16)
C(15)-C(14)-H(14A)	119.7
C(13)-C(14)-H(14A)	119.7
C(14)-C(15)-C(16)	121.14(14)
C(14)-C(15)-H(15A)	119.4
C(16)-C(15)-H(15A)	119.4
C(15)-C(16)-C(11)	118.09(13)
C(15)-C(16)-C(17)	122.54(12)
C(11)-C(16)-C(17)	119.34(13)
C(8)-C(17)-C(16)	118.17(12)
C(8)-C(17)-C(18)	120.28(12)
C(16)-C(17)-C(18)	121.55(12)
N(1)-C(18)-C(19)	122.43(12)
N(1)-C(18)-C(17)	116.50(11)
C(19)-C(18)-C(17)	121.08(12)
C(18)-C(19)-H(19A)	120.2
C(20)-C(19)-H(19A)	120.2
C(21)-C(20)-C(19)	118.81(15)
C(21)-C(20)-H(20A)	120.6
C(19)-C(20)-H(20A)	120.6
C(22)-C(21)-C(20)	118.28(15)
C(22)-C(21)-H(21A)	120.9
C(20)-C(21)-H(21A)	120.9
N(1)-C(22)-C(21)	124.68(15)
N(1)-C(22)-H(22A)	117.7
C(21)-C(22)-H(22A)	117.7

Symmetry transformations used to generate equivalent atoms.

Table S4. Anisotropic displacement parameters ($Å^2 \times 10^3$) for **2i**. The anisotropic displacement factor exponent takes the form: $-2\pi^2[h^2a^{*2}U^{11} + ... + 2 h k a^* b^* U^{12}]$.

	U^{11}	U^{22}	U^{33}	U^{23}	U^{13}	U^{12}
O(1)	60(1)	73(1)	68(1)	0(1)	−27(1)	−7(1)
O(2)	56(1)	95(1)	81(1)	8(1)	−22(1)	−9(1)
N(1)	74(1)	56(1)	125(1)	2(1)	−49(1)	−9(1)
C(1)	54(1)	57(1)	62(1)	−6(1)	−25(1)	−14(1)
C(2)	62(1)	65(1)	80(1)	−5(1)	−25(1)	−10(1)
C(3)	75(1)	86(1)	74(1)	−5(1)	−12(1)	−19(1)
C(4)	93(1)	79(1)	78(1)	13(1)	−26(1)	−24(1)
C(5)	83(1)	70(1)	92(1)	12(1)	−36(1)	−12(1)
C(6)	63(1)	66(1)	76(1)	−5(1)	−26(1)	−9(1)
C(7)	54(1)	63(1)	69(1)	−9(1)	−30(1)	−9(1)
C(8)	58(1)	60(1)	69(1)	0(1)	−27(1)	−6(1)
C(9)	75(1)	66(1)	90(1)	−12(1)	−38(1)	−13(1)
C(10)	77(1)	52(1)	109(1)	−1(1)	−40(1)	−15(1)
C(11)	58(1)	49(1)	88(1)	4(1)	−28(1)	−4(1)
C(12)	71(1)	55(1)	110(1)	19(1)	−32(1)	−8(1)
C(13)	78(1)	76(1)	95(1)	26(1)	−31(1)	−3(1)
C(14)	79(1)	87(1)	83(1)	15(1)	−38(1)	−4(1)
C(15)	65(1)	67(1)	77(1)	9(1)	−32(1)	−9(1)
C(16)	48(1)	51(1)	73(1)	2(1)	−24(1)	−1(1)
C(17)	48(1)	51(1)	68(1)	−1(1)	−24(1)	−3(1)
C(18)	53(1)	57(1)	61(1)	2(1)	−25(1)	−11(1)
C(19)	54(1)	80(1)	91(1)	6(1)	−29(1)	−13(1)
C(20)	69(1)	117(1)	95(1)	6(1)	−32(1)	−41(1)
C(21)	109(1)	88(1)	93(1)	12(1)	−45(1)	−53(1)
C(22)	110(1)	59(1)	144(2)	8(1)	−64(1)	−20(1)

Table S5. Hydrogen coordinates ($\times 10^4$) and isotropic displacement parameters ($Å^2 \times 10^3$) for **2i**.

	x	y	z	U(eq)
H(2A)	9244	6895	2489	83
H(3A)	9898	8071	395	99
H(4A)	8076	9855	101	101
H(5A)	5570	10481	1864	98
H(6A)	4871	9294	3945	82
H(9A)	6277	4195	5518	89
H(10A)	5805	2855	7462	93
H(12A)	5888	2381	9820	97
H(13A)	6529	2947	11634	104
H(14A)	7807	4833	11467	99
H(15A)	8386	6153	9507	82
H(19A)	10793	6170	6813	90
H(20A)	11957	8120	6539	109
H(21A)	10369	10240	6637	109
H(22A)	7739	10338	6966	119

Table S6. Torsion angles [°] for **2i**.

C(6)-C(1)-C(2)-C(3)	−0.9(2)
C(7)-C(1)-C(2)-C(3)	177.30(13)
C(1)-C(2)-C(3)-C(4)	1.0(2)
C(2)-C(3)-C(4)-C(5)	−0.4(3)
C(3)-C(4)-C(5)-C(6)	−0.3(3)
C(4)-C(5)-C(6)-C(1)	0.4(2)
C(2)-C(1)-C(6)-C(5)	0.2(2)
C(7)-C(1)-C(6)-C(5)	−178.08(13)
C(8)-O(1)-C(7)-O(2)	−1.70(19)
C(8)-O(1)-C(7)-C(1)	179.57(10)
C(2)-C(1)-C(7)-O(2)	−168.00(13)
C(6)-C(1)-C(7)-O(2)	10.2(2)
C(2)-C(1)-C(7)-O(1)	10.69(18)
C(6)-C(1)-C(7)-O(1)	−171.09(11)
C(7)-O(1)-C(8)-C(17)	−105.10(13)
C(7)-O(1)-C(8)-C(9)	79.31(14)
C(17)-C(8)-C(9)-C(10)	0.6(2)
O(1)-C(8)-C(9)-C(10)	176.01(12)
C(8)-C(9)-C(10)-C(11)	−0.1(2)
C(9)-C(10)-C(11)-C(12)	177.21(13)
C(9)-C(10)-C(11)-C(16)	−0.3(2)
C(10)-C(11)-C(12)-C(13)	−177.85(13)
C(16)-C(11)-C(12)-C(13)	−0.4(2)
C(11)-C(12)-C(13)-C(14)	−0.3(2)
C(12)-C(13)-C(14)-C(15)	0.6(2)
C(13)-C(14)-C(15)-C(16)	−0.2(2)
C(14)-C(15)-C(16)-C(11)	−0.45(19)
C(14)-C(15)-C(16)-C(17)	177.66(12)
C(10)-C(11)-C(16)-C(15)	178.30(12)
C(12)-C(11)-C(16)-C(15)	0.74(18)
C(10)-C(11)-C(16)-C(17)	0.12(18)
C(12)-C(11)-C(16)-C(17)	−177.43(11)
C(9)-C(8)-C(17)-C(16)	−0.77(19)
O(1)-C(8)-C(17)-C(16)	−176.12(10)
C(9)-C(8)-C(17)-C(18)	179.75(12)
O(1)-C(8)-C(17)-C(18)	4.40(17)
C(15)-C(16)-C(17)-C(8)	−177.71(11)
C(11)-C(16)-C(17)-C(8)	0.38(17)
C(15)-C(16)-C(17)-C(18)	1.77(18)
C(11)-C(16)-C(17)-C(18)	179.85(11)
C(22)-N(1)-C(18)-C(19)	0.6(2)
C(22)-N(1)-C(18)-C(17)	−179.15(14)
C(8)-C(17)-C(18)-N(1)	68.44(16)
C(16)-C(17)-C(18)-N(1)	−111.02(14)
C(8)-C(17)-C(18)-C(19)	−111.35(15)
C(16)-C(17)-C(18)-C(19)	69.19(17)
N(1)-C(18)-C(19)-C(20)	−0.6(2)
C(17)-C(18)-C(19)-C(20)	179.15(14)
C(18)-C(19)-C(20)-C(21)	0.5(2)
C(19)-C(20)-C(21)-C(22)	−0.4(3)
C(18)-N(1)-C(22)-C(21)	−0.5(3)
C(20)-C(21)-C(22)-N(1)	0.4(3)

Symmetry transformations used to generate equivalent atoms.

2. ¹H and ¹³C-NMR Spectra:

¹H-NMR of **2a**

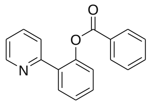

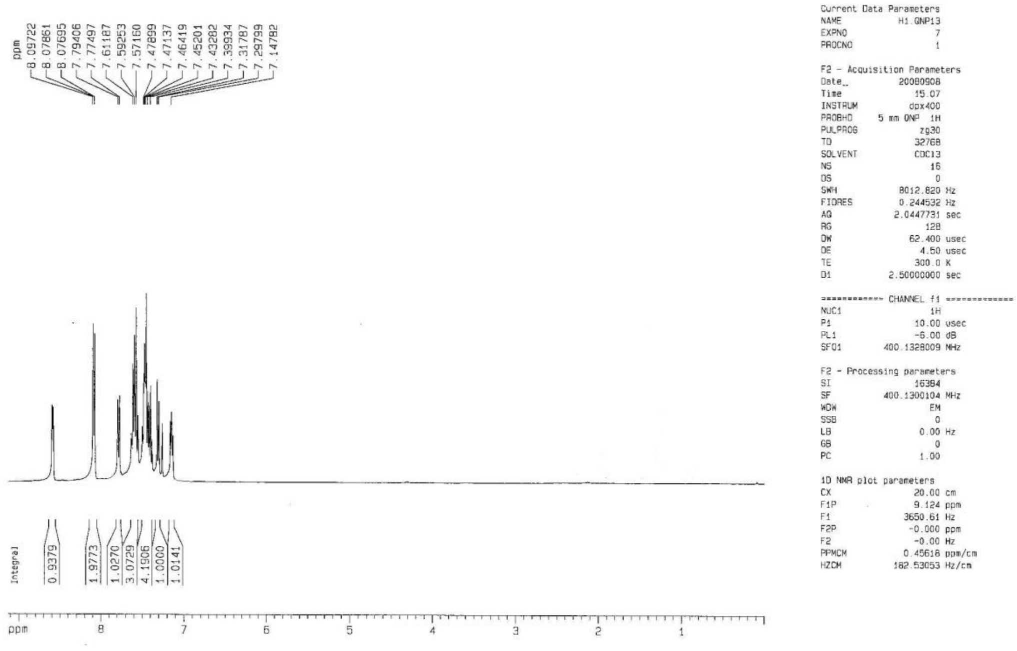

¹³C-NMR of **2a**

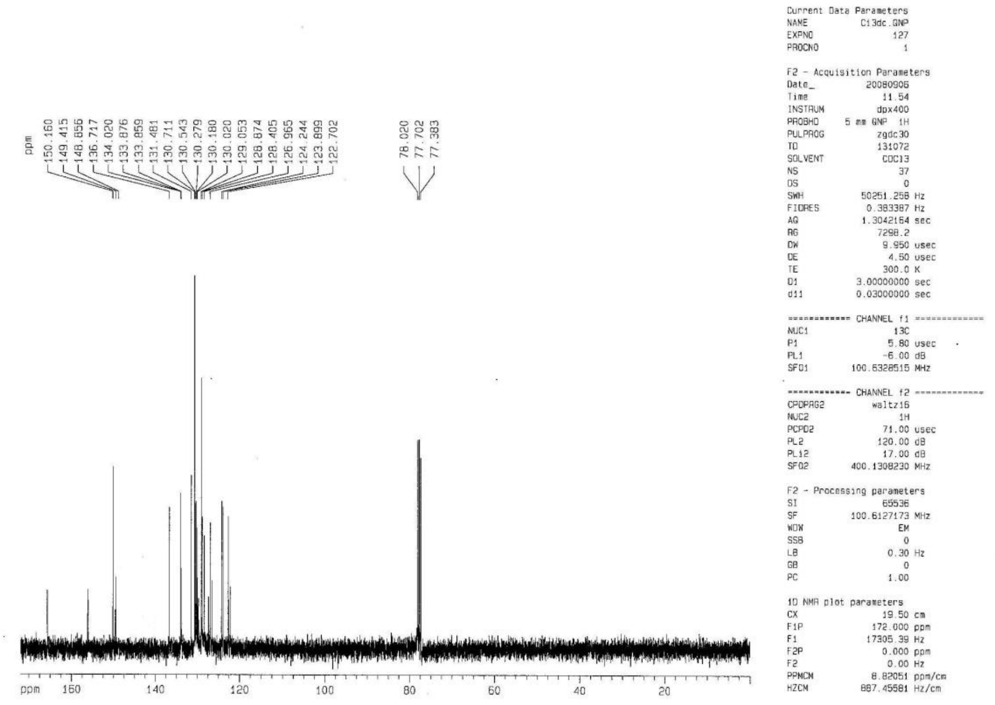

^{1}H-NMR of **2b**

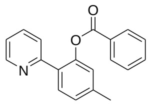

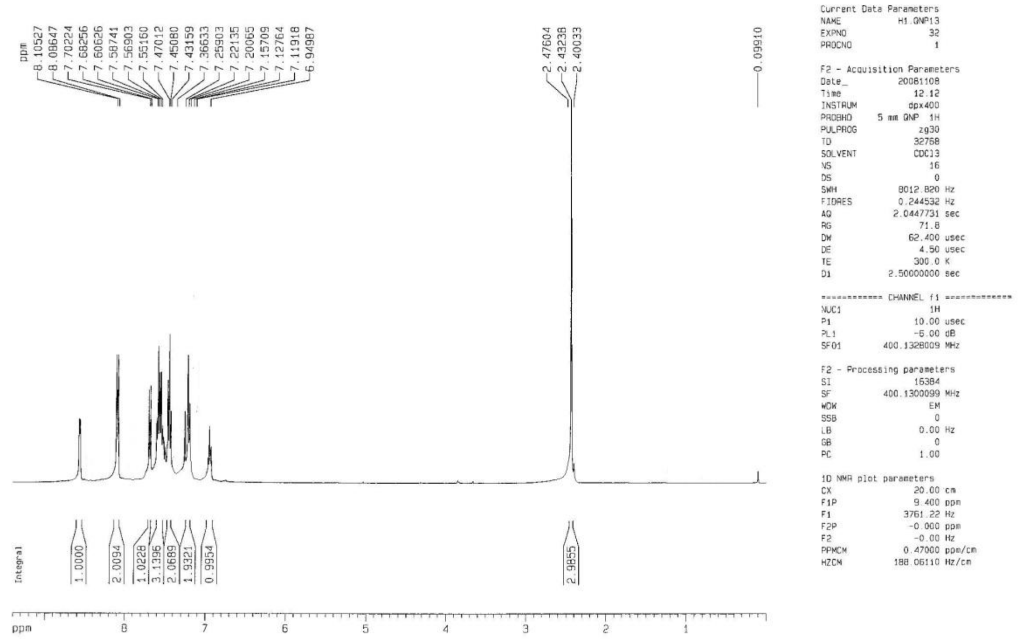

^{13}C-NMR of **2b**

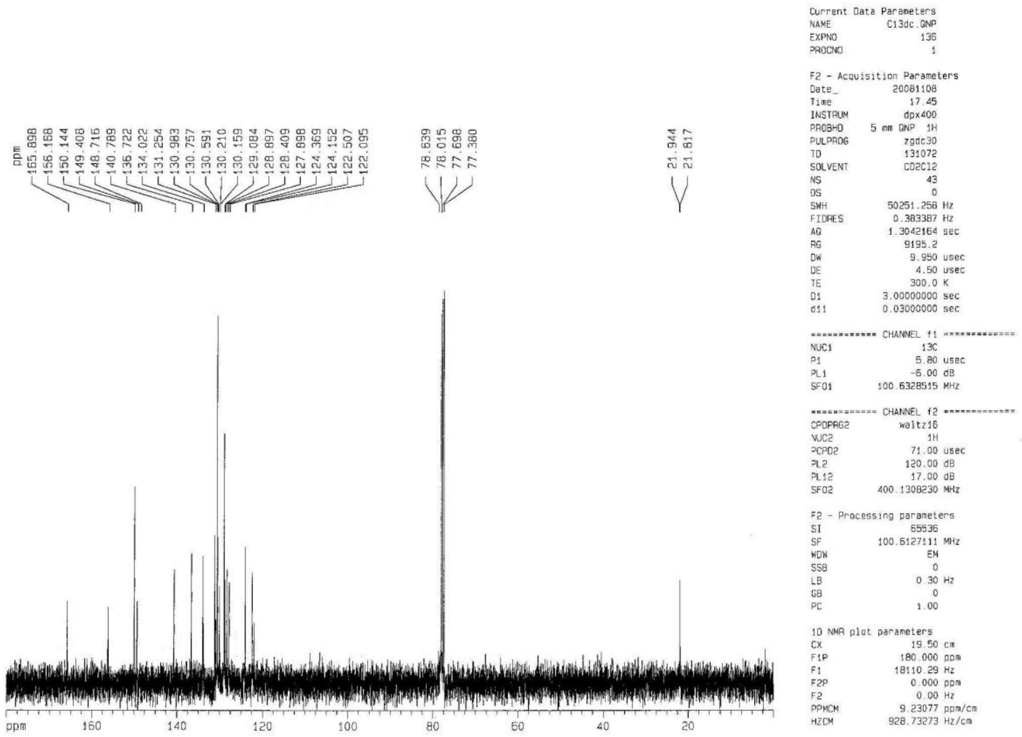

^{1}H-NMR of **2c**

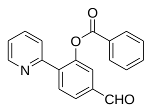

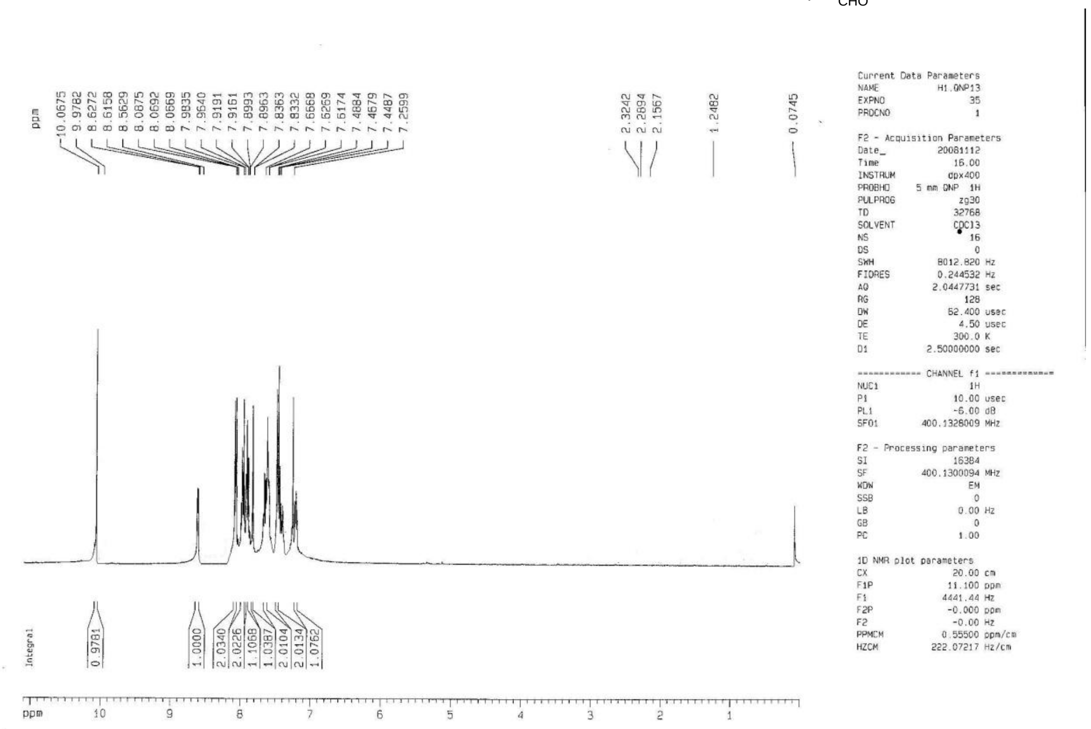

^{13}C-NMR of **2c**

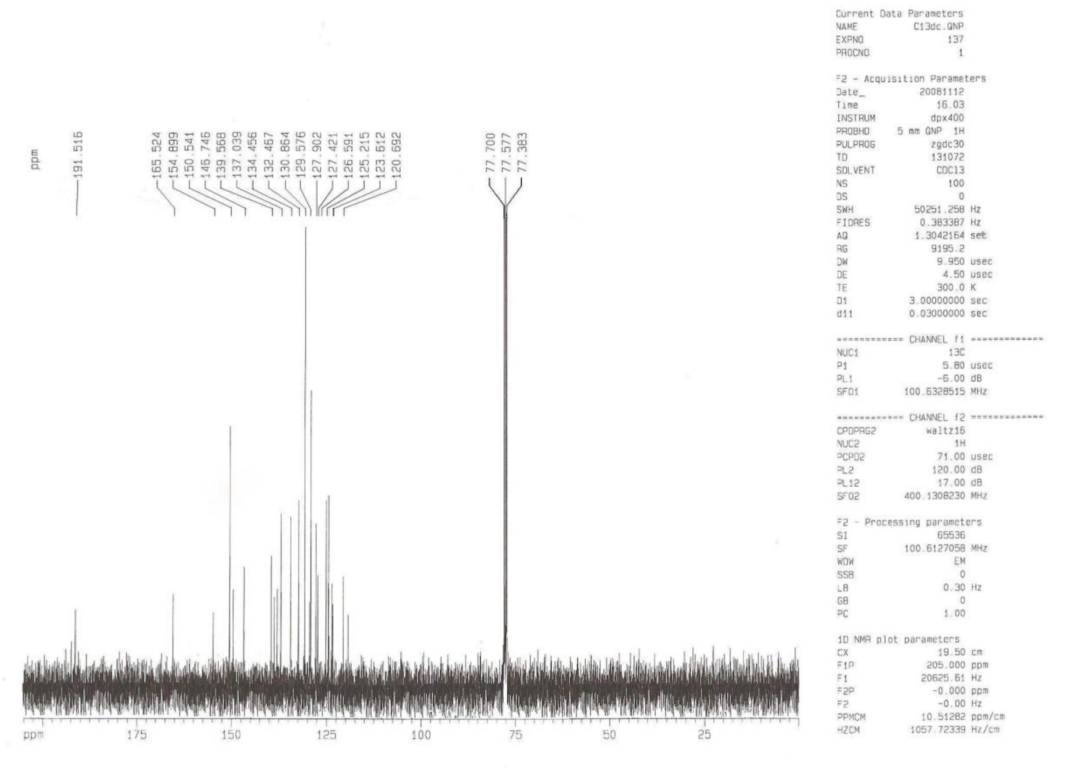

^{1}H-NMR of **2d**

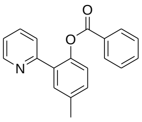

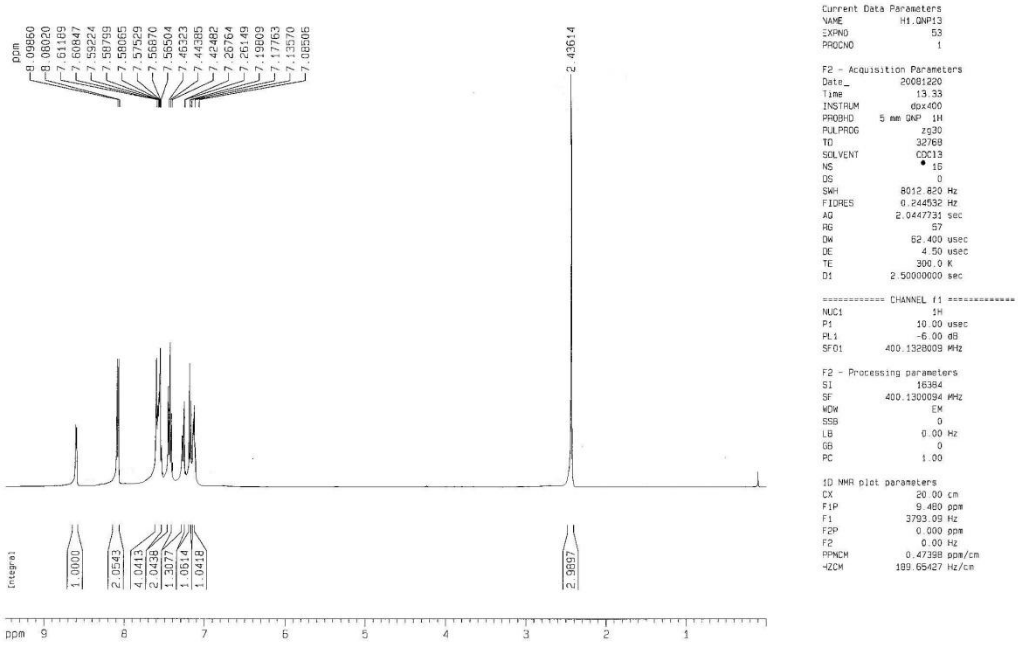

^{13}C-NMR of **2d**

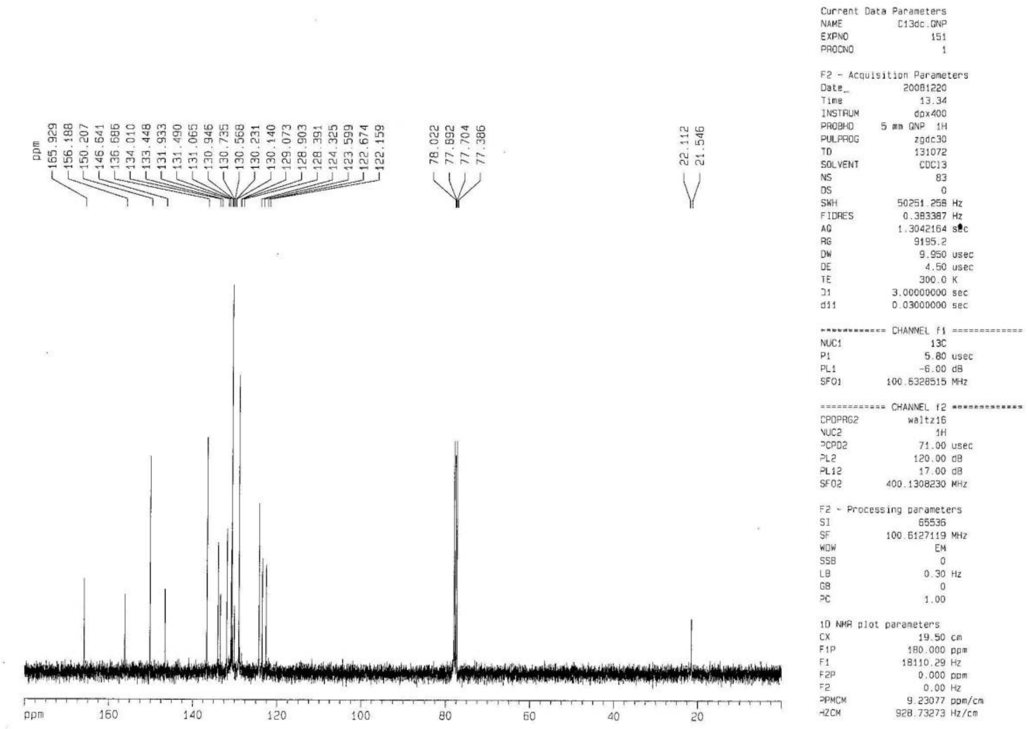

^{1}H-NMR of **2e**

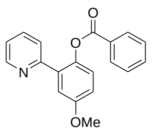

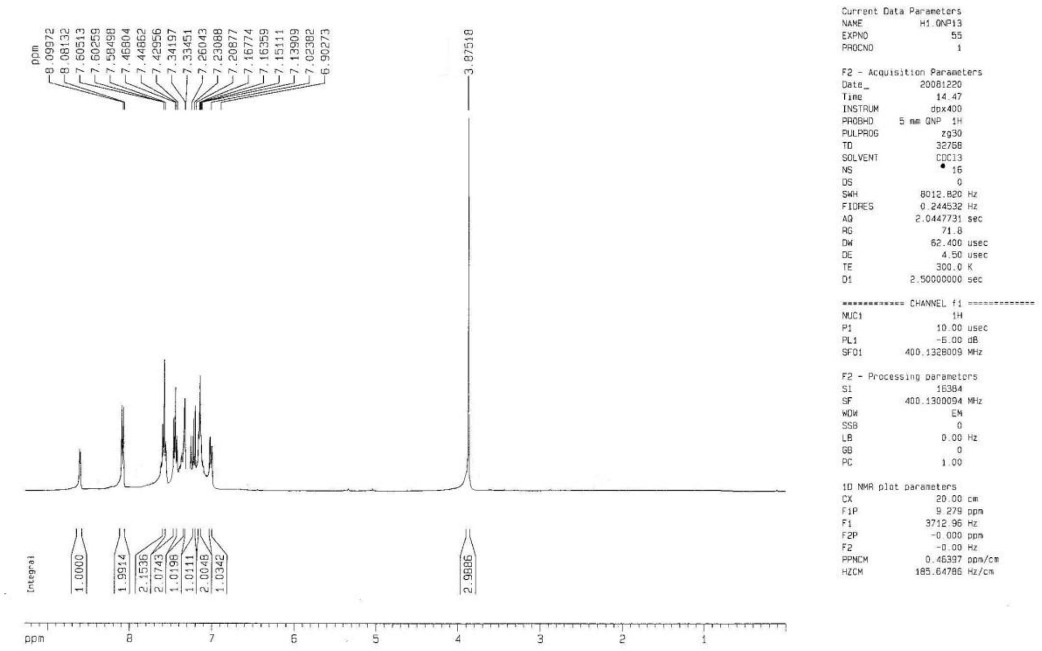

^{13}C-NMR of **2e**

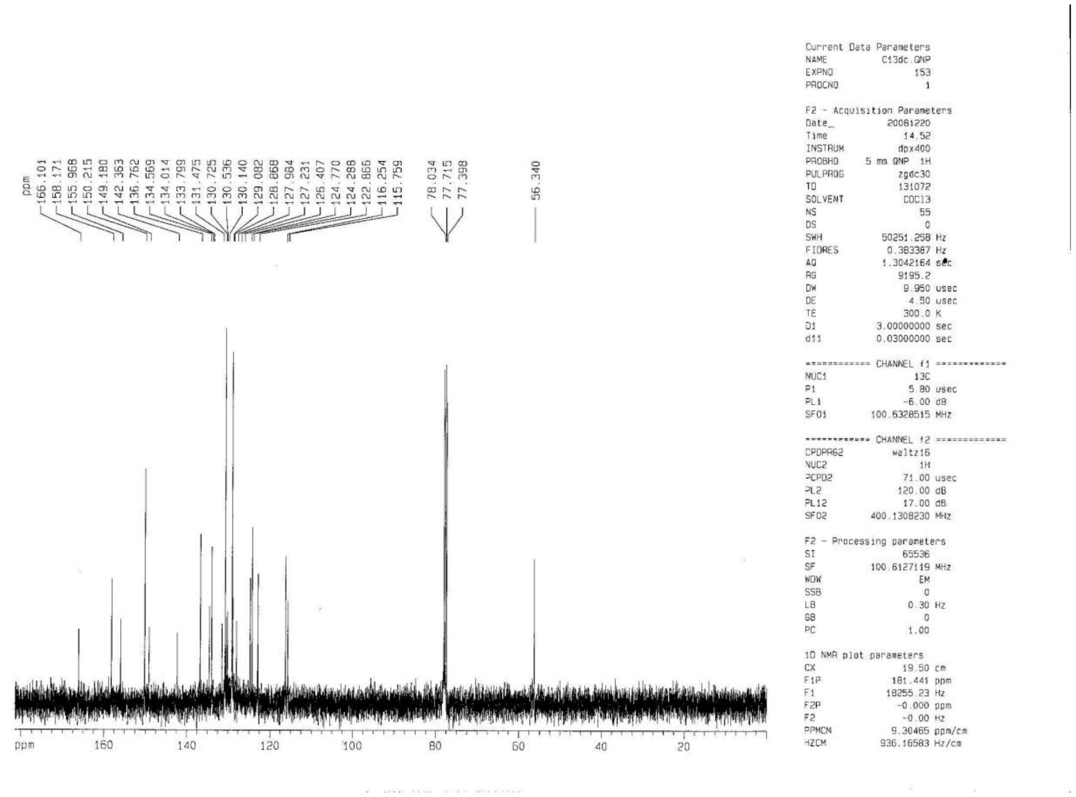

^{1}H-NMR of **2f**

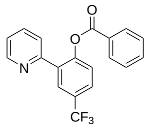

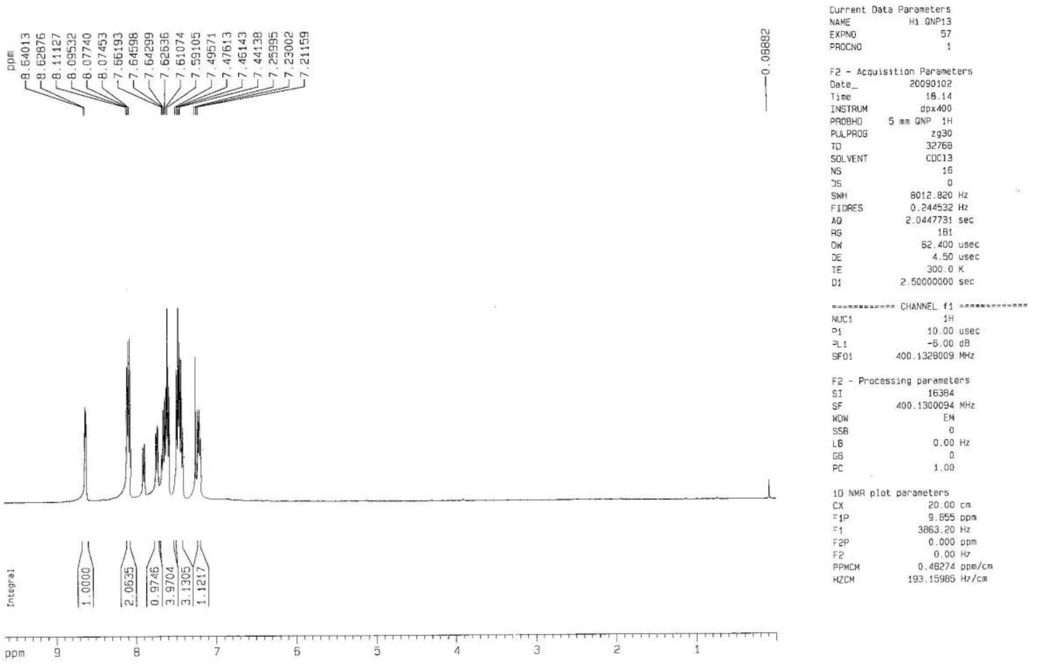

^{13}C-NMR of **2f**

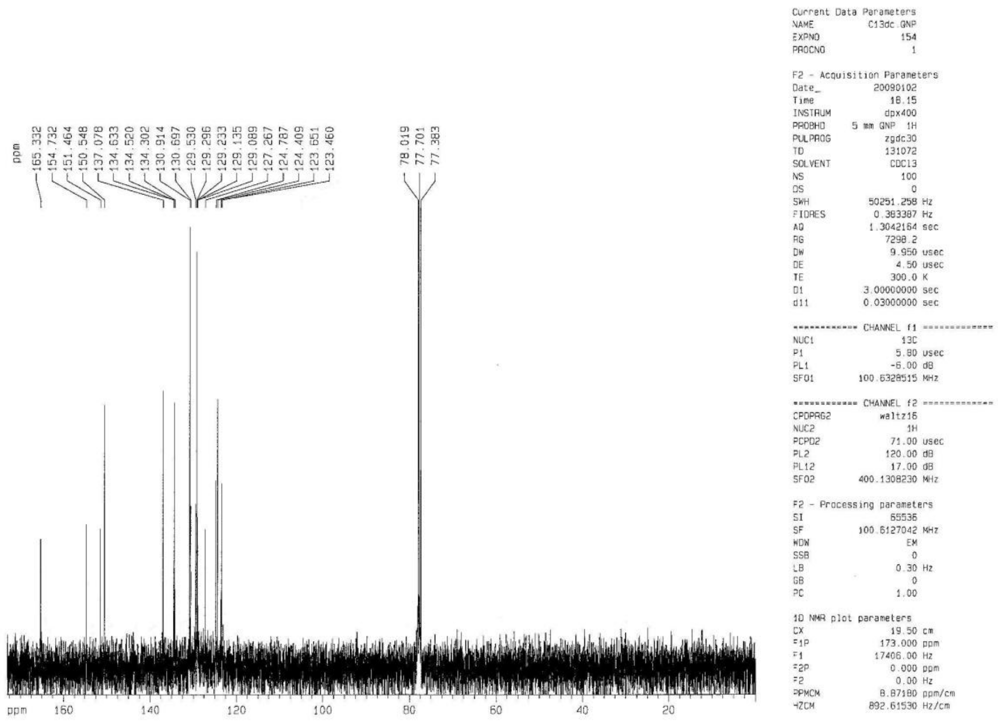

^{1}H-NMR of **2g**

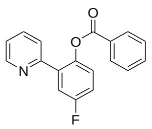

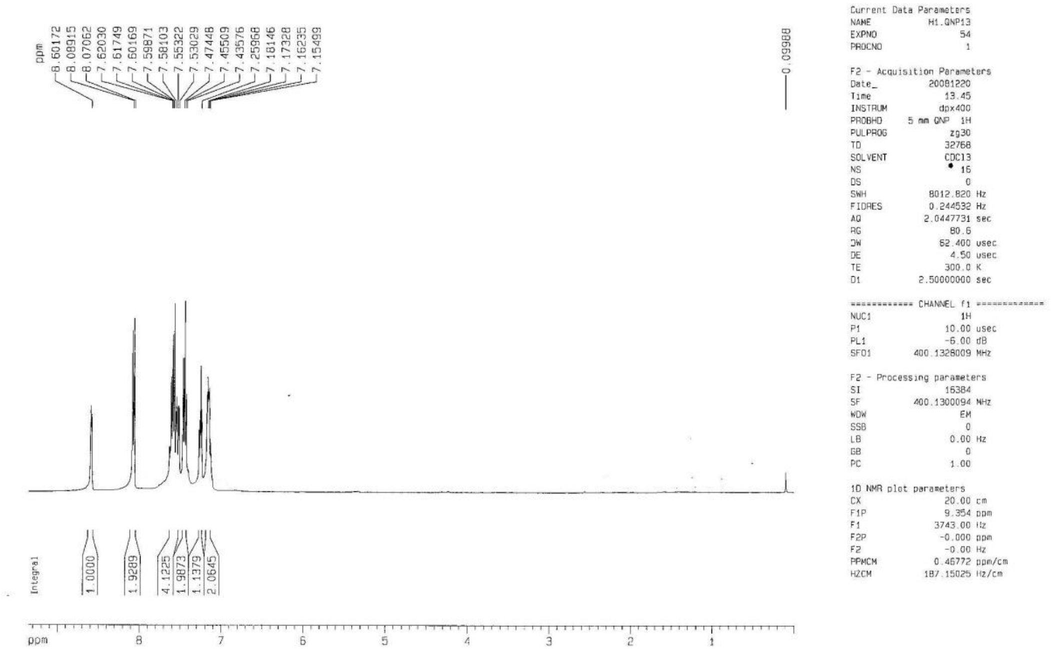

^{13}C-NMR of **2g**

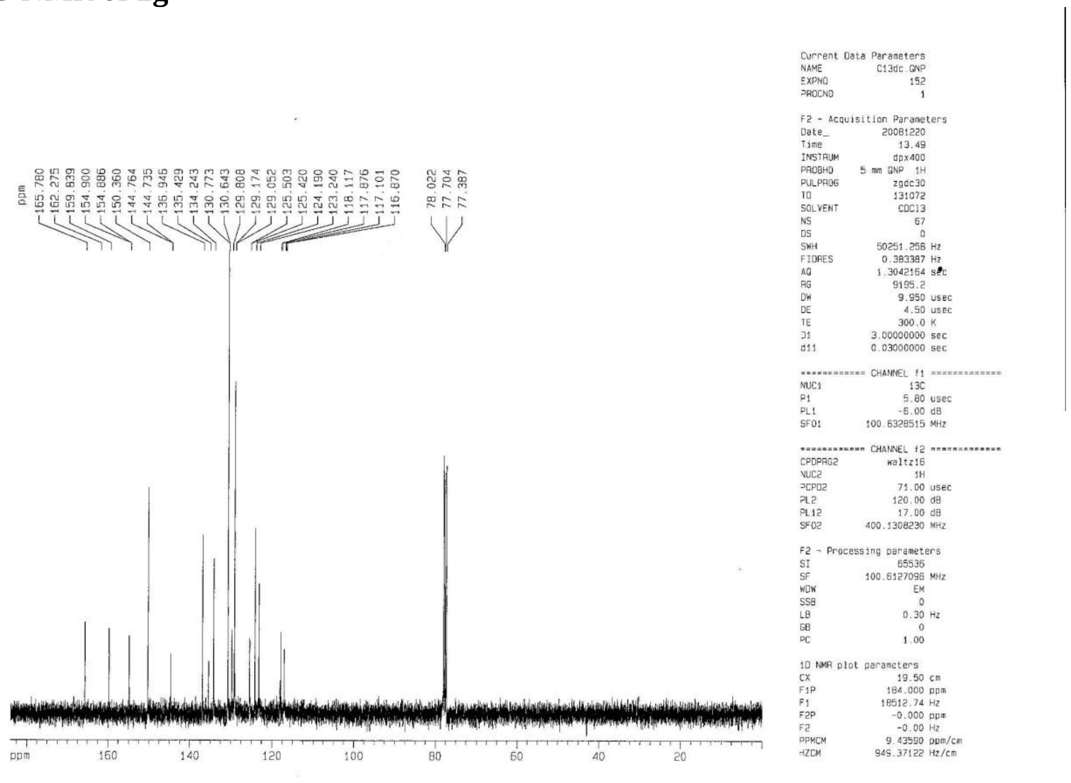

^{1}H-NMR of **2h**

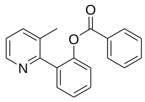

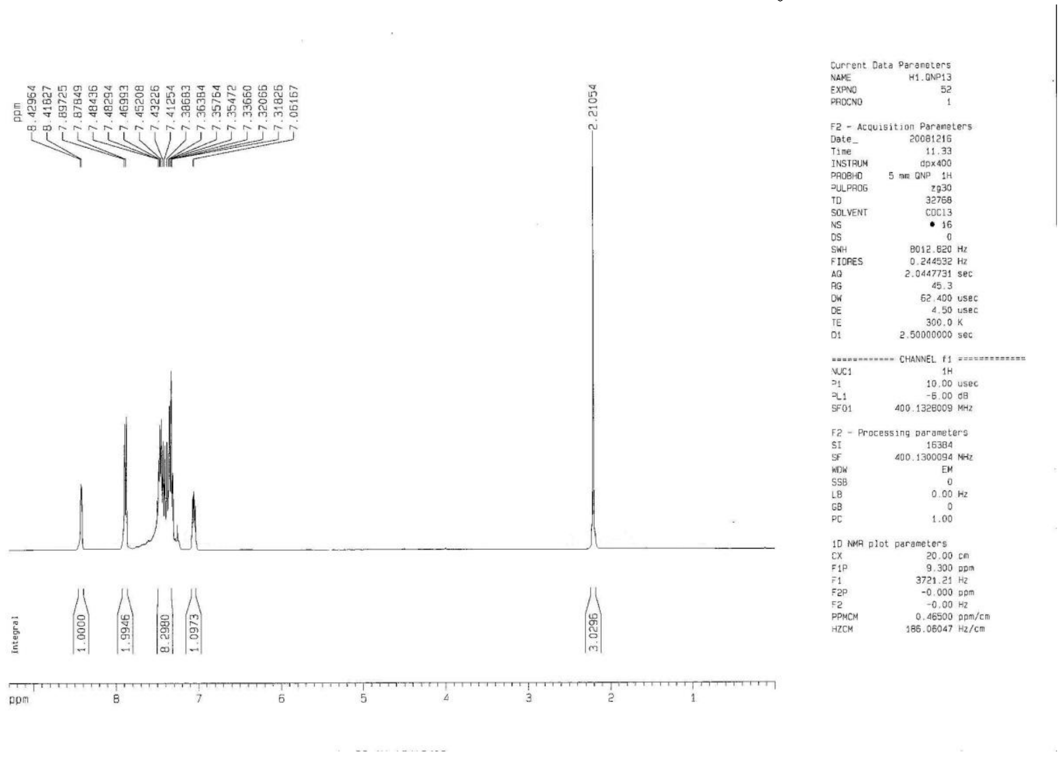

^{13}C-NMR of **2h**

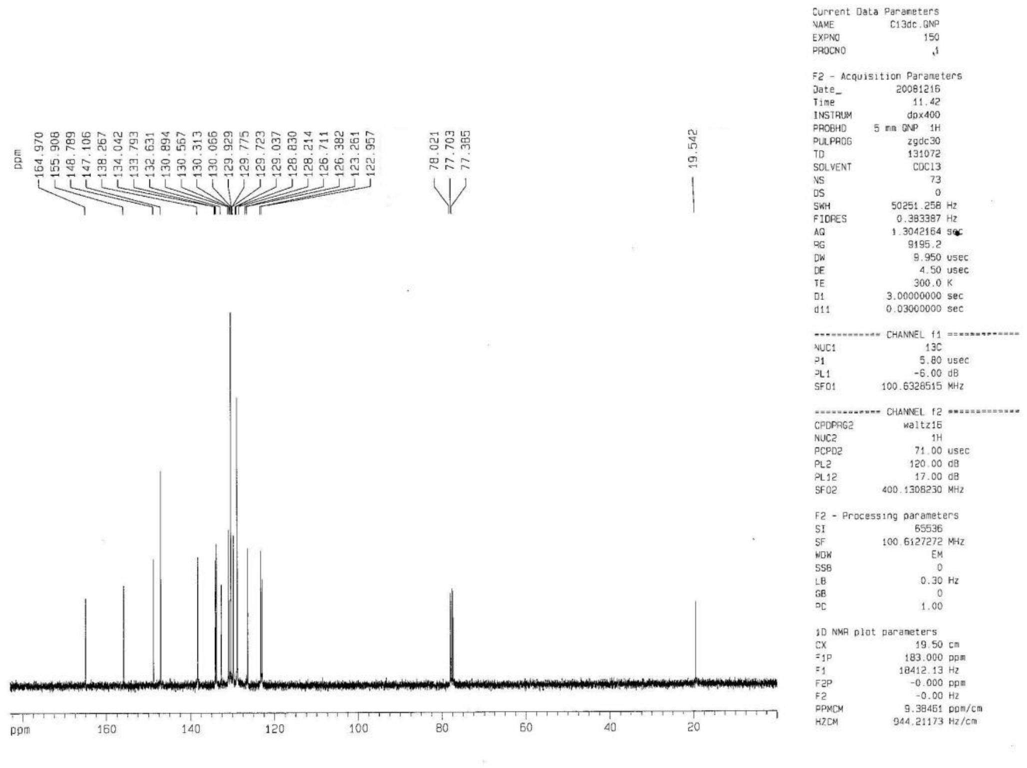

^{1}H-NMR of **2i**

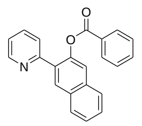

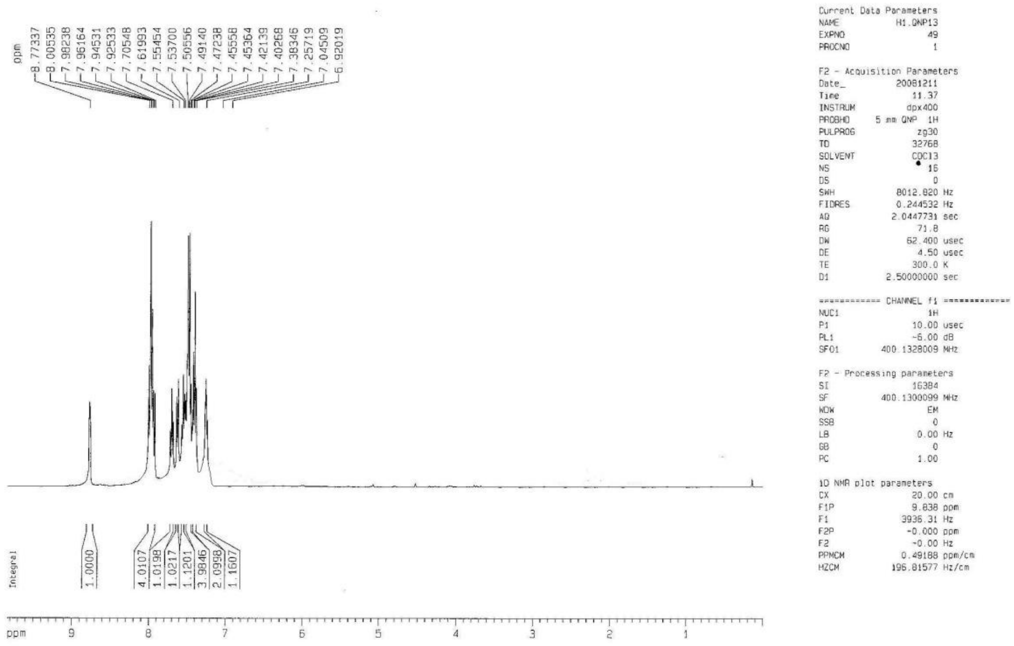

^{13}C-NMR of **2i**

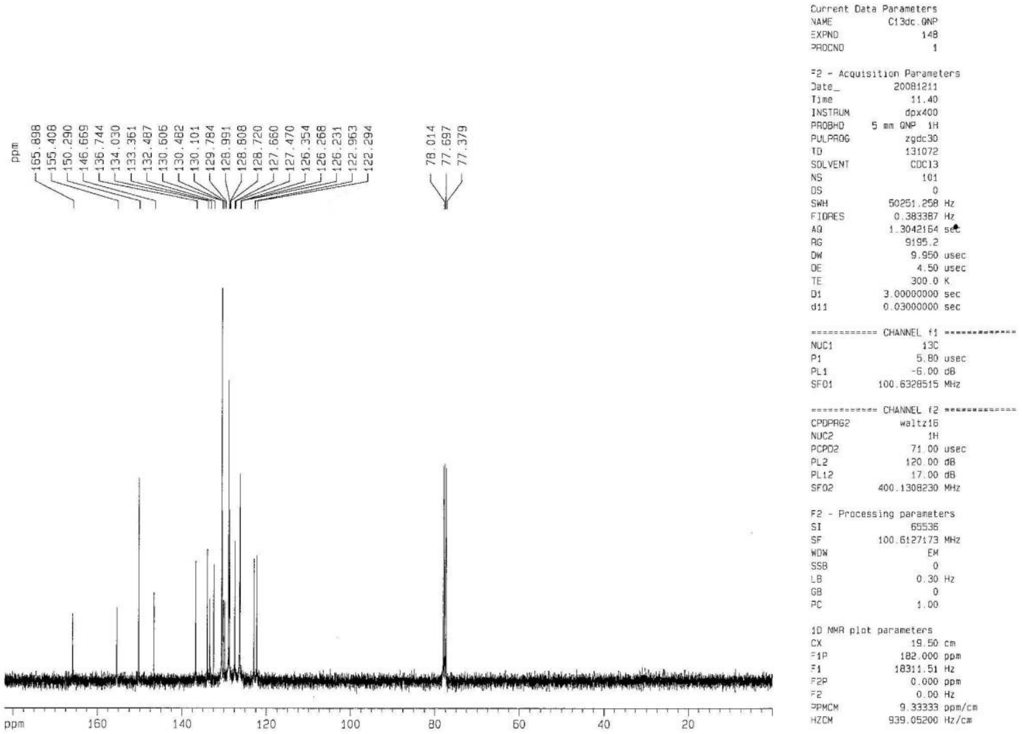

^{1}H-NMR of **2j**

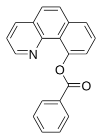

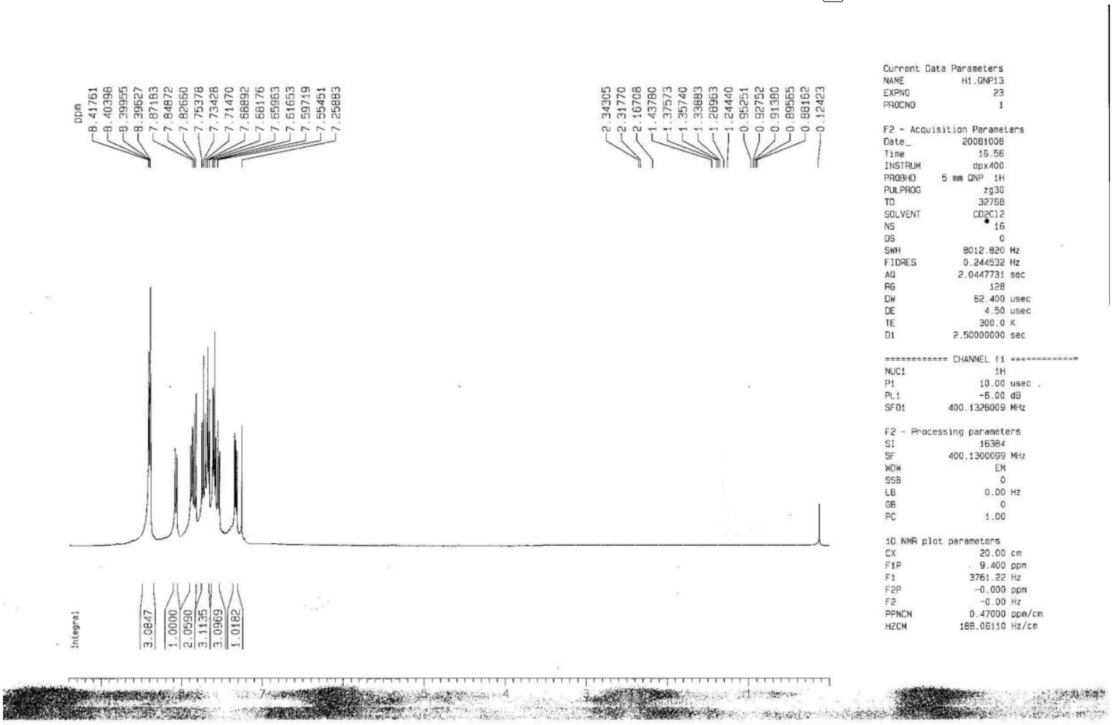

^{13}C-NMR of **2j**

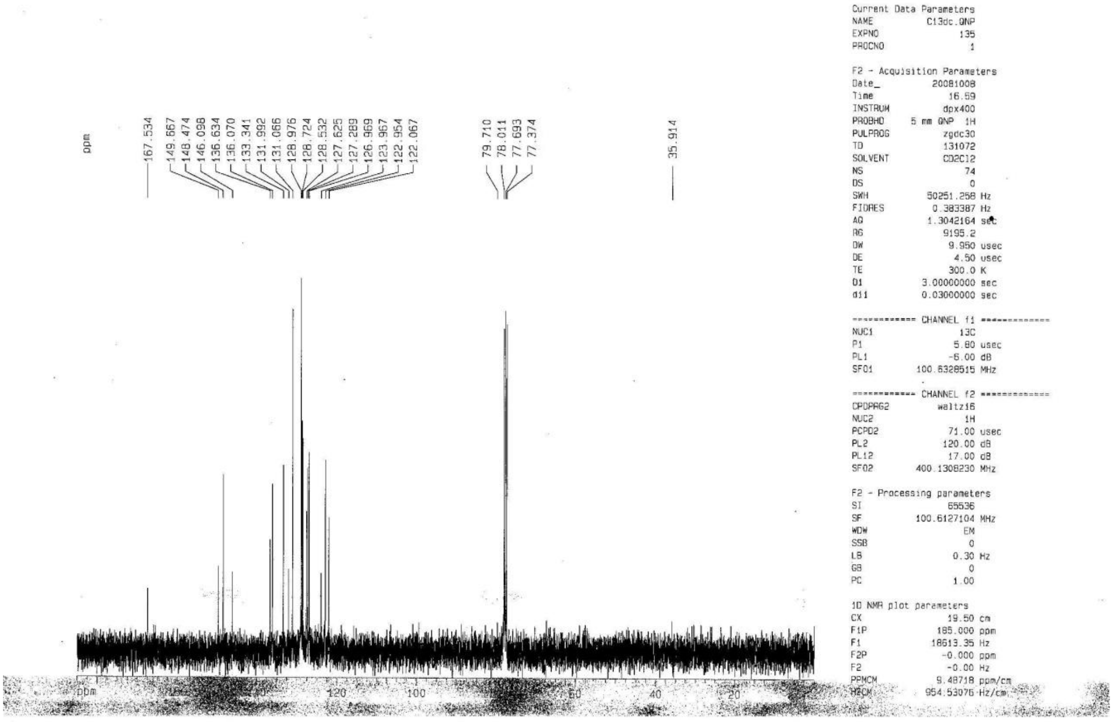

^{1}H-NMR of **2k**

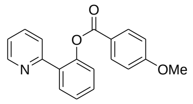

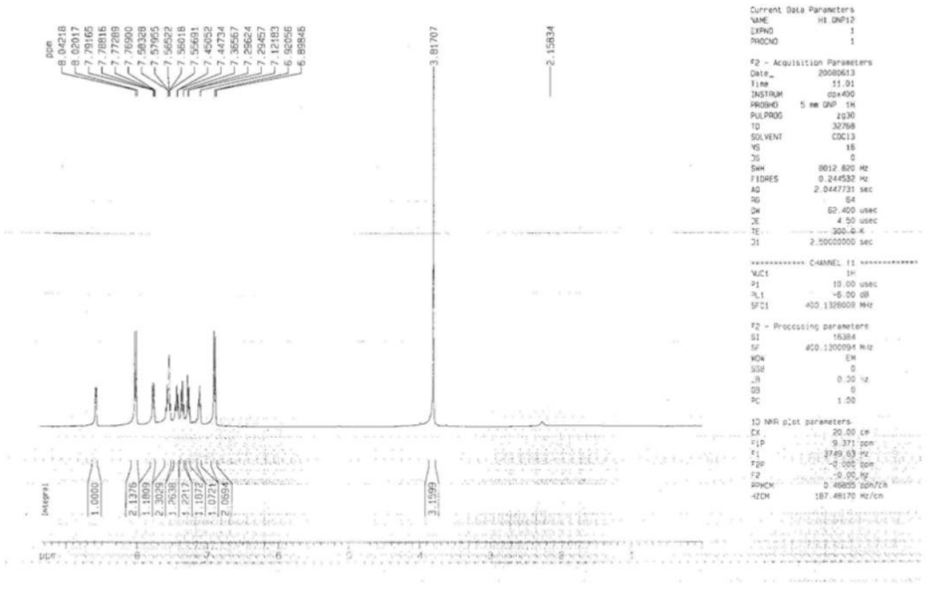

^{13}C-NMR of **2k**

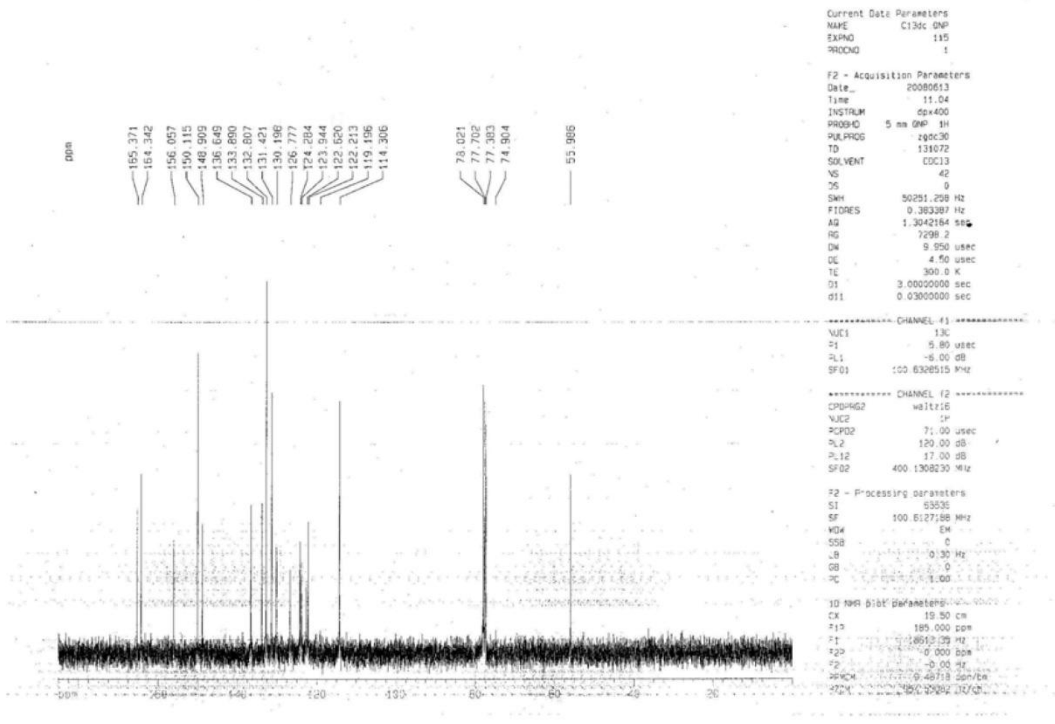

^{1}H-NMR of **2l**

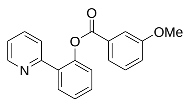

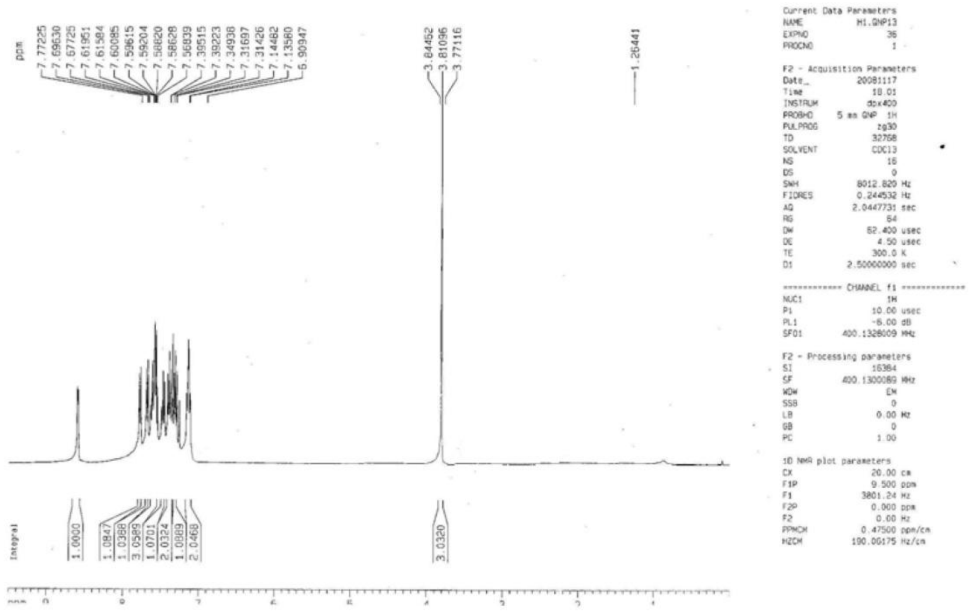

^{13}C-NMR of **2l**

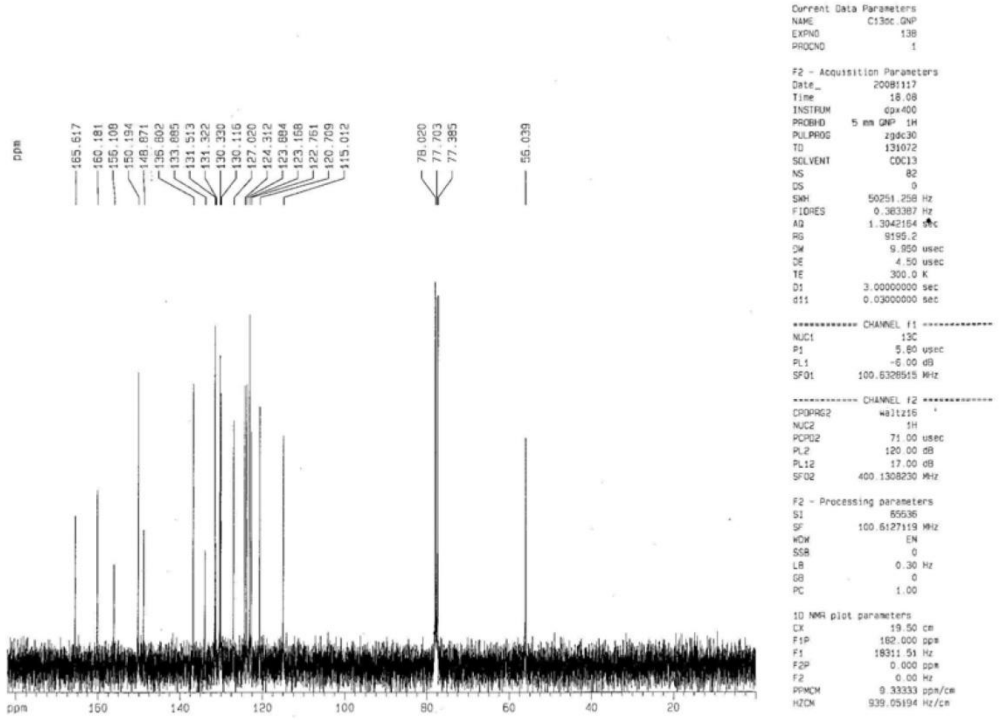

^{1}H-NMR of **2m**

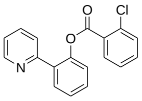

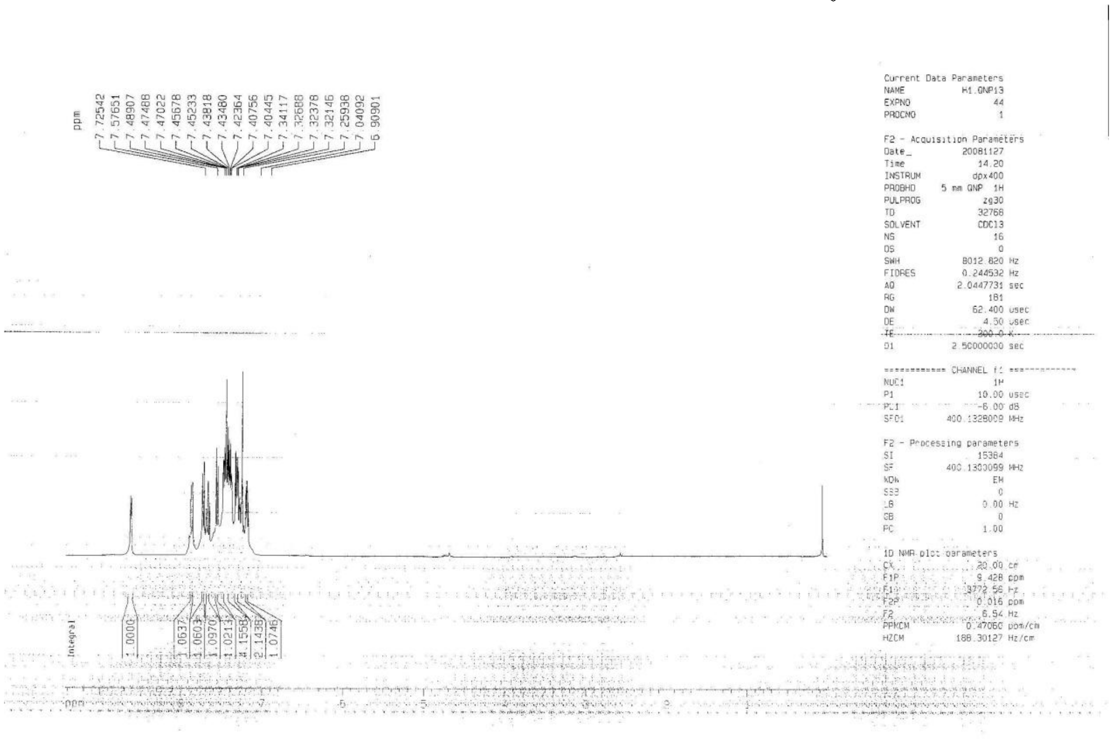

^{13}C-NMR of **2m**

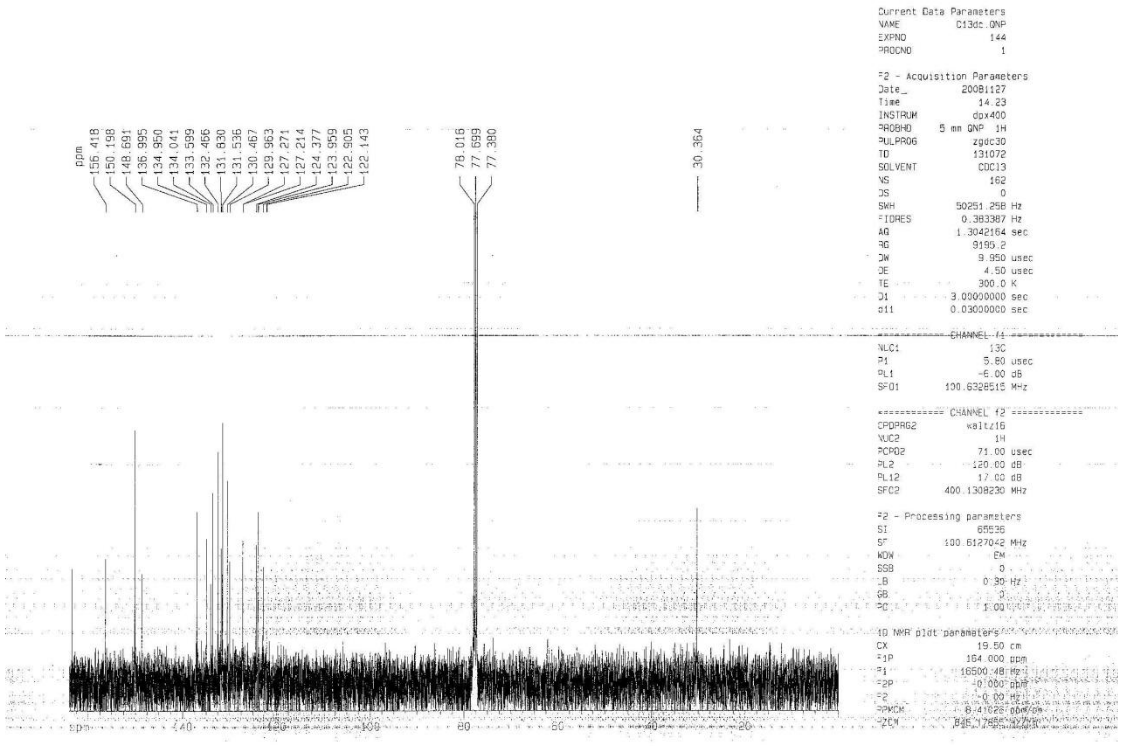

^{1}H-NMR of **2n**

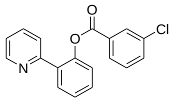

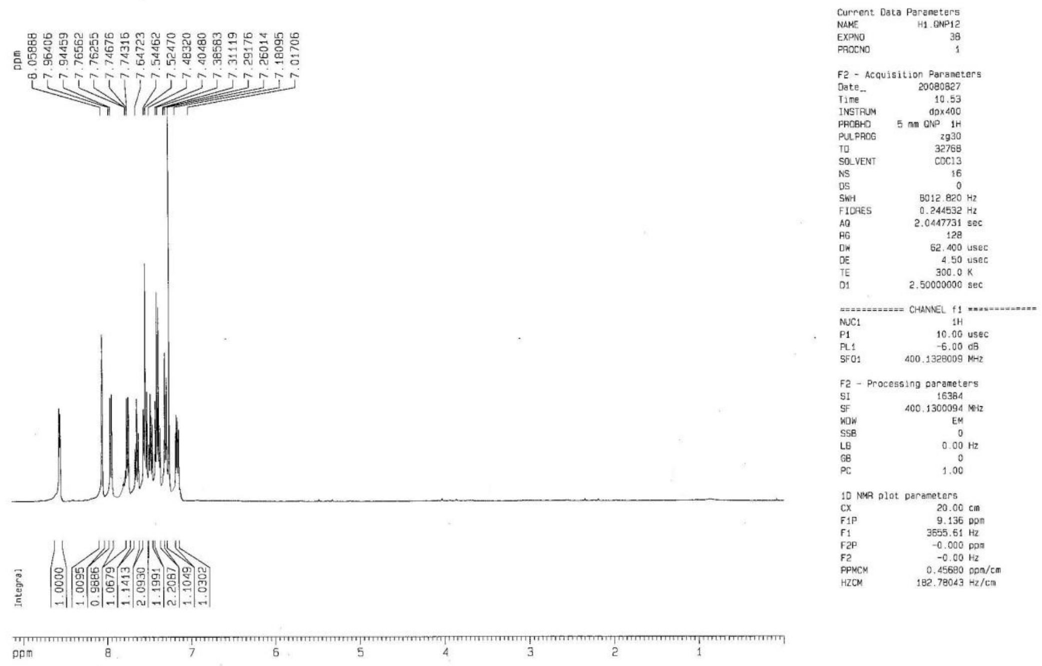

^{13}C-NMR of **2n**

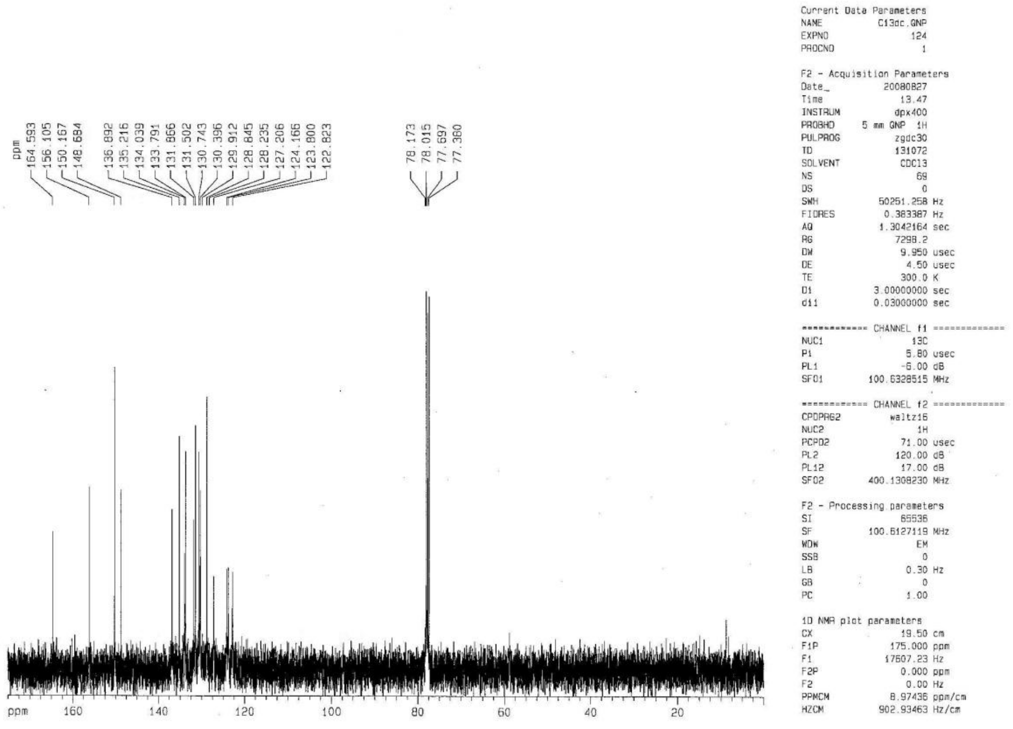

^{1}H-NMR of **2o**

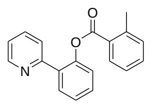

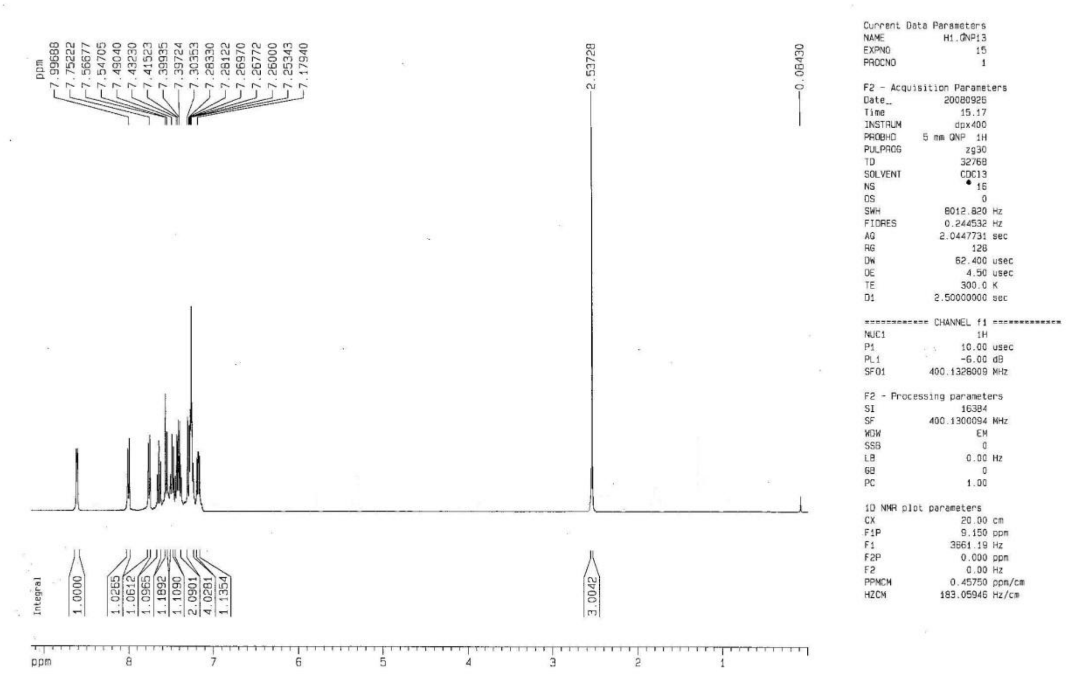

^{13}C-NMR of **2o**

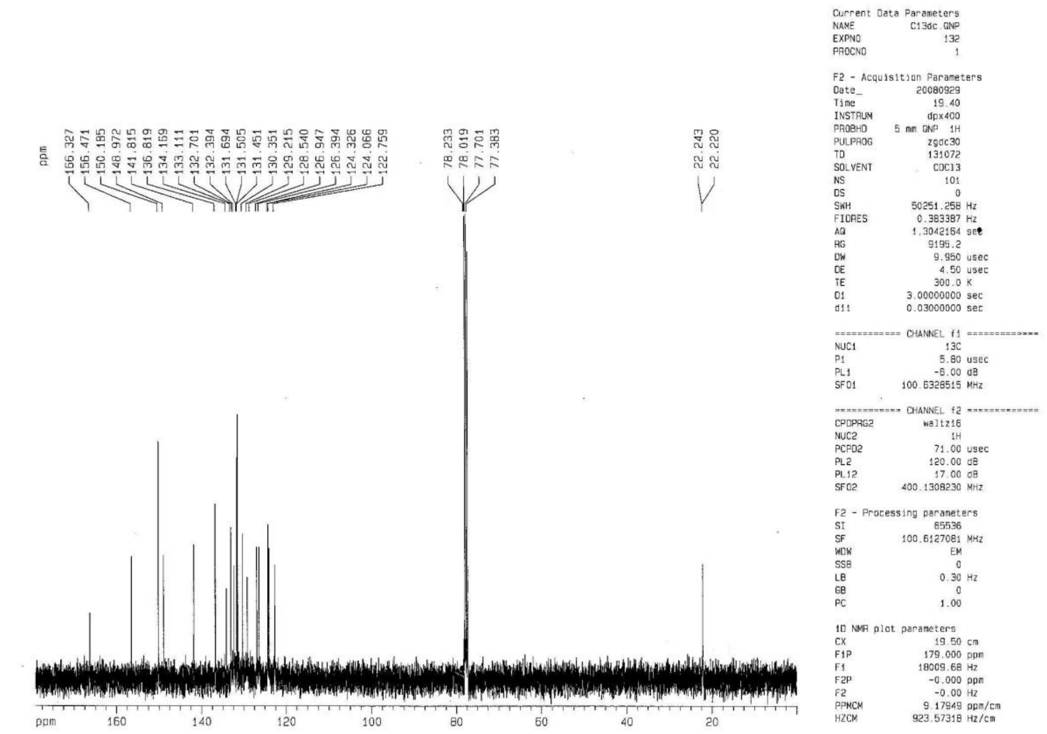

^{1}H-NMR of **2p**

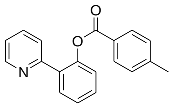

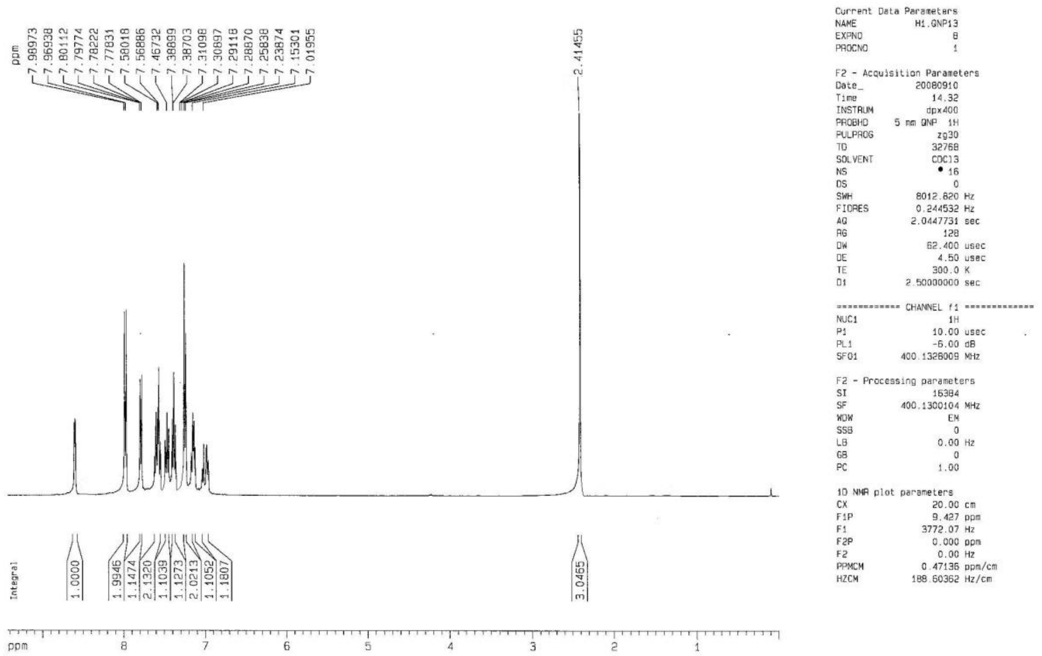

^{13}C-NMR of **2p**

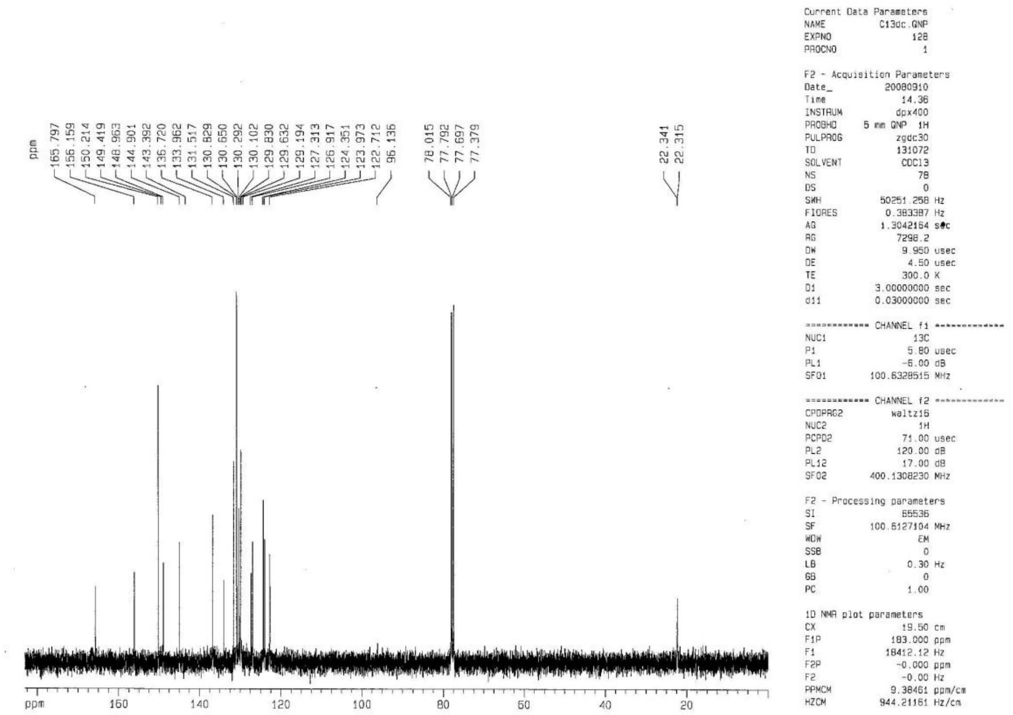

^{1}H-NMR of **2q**

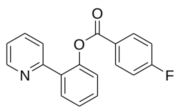

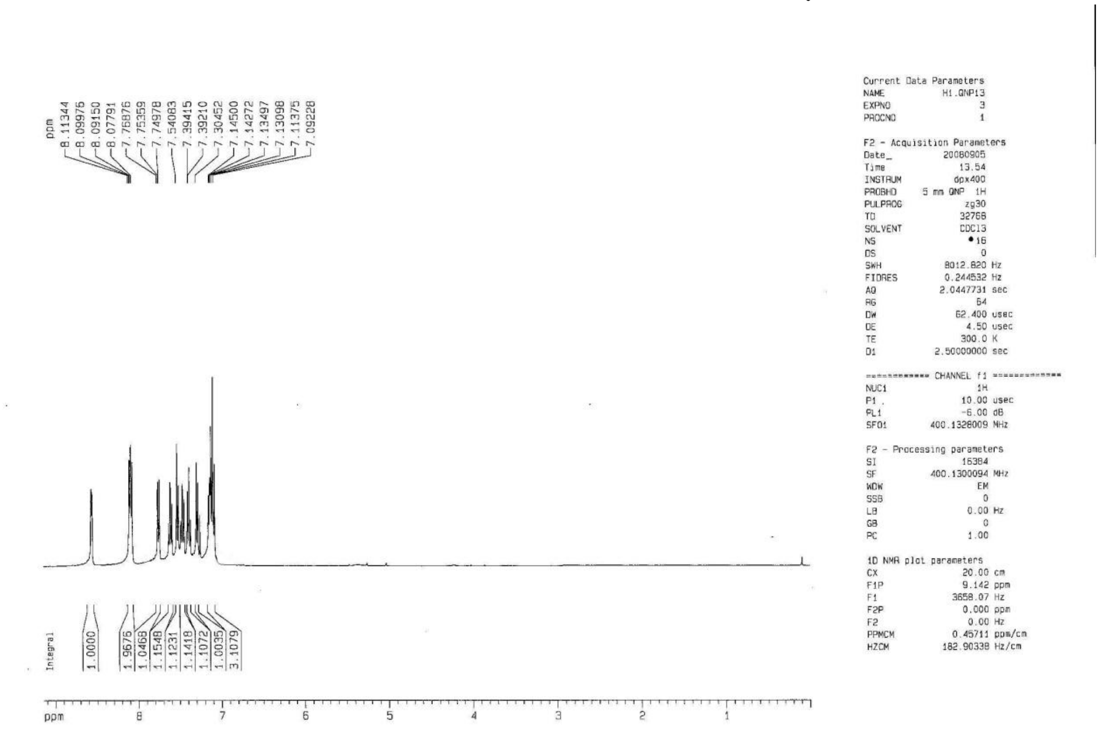

^{13}C-NMR of **2q**

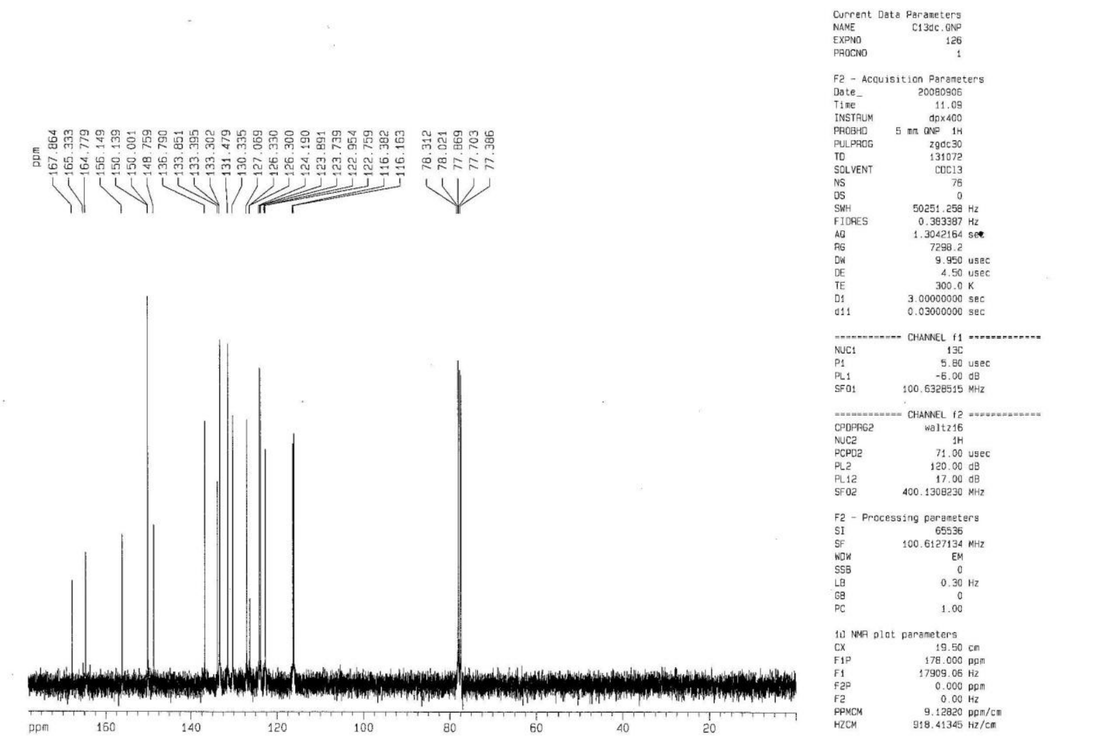

^{1}H-NMR of **2r**

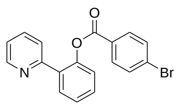

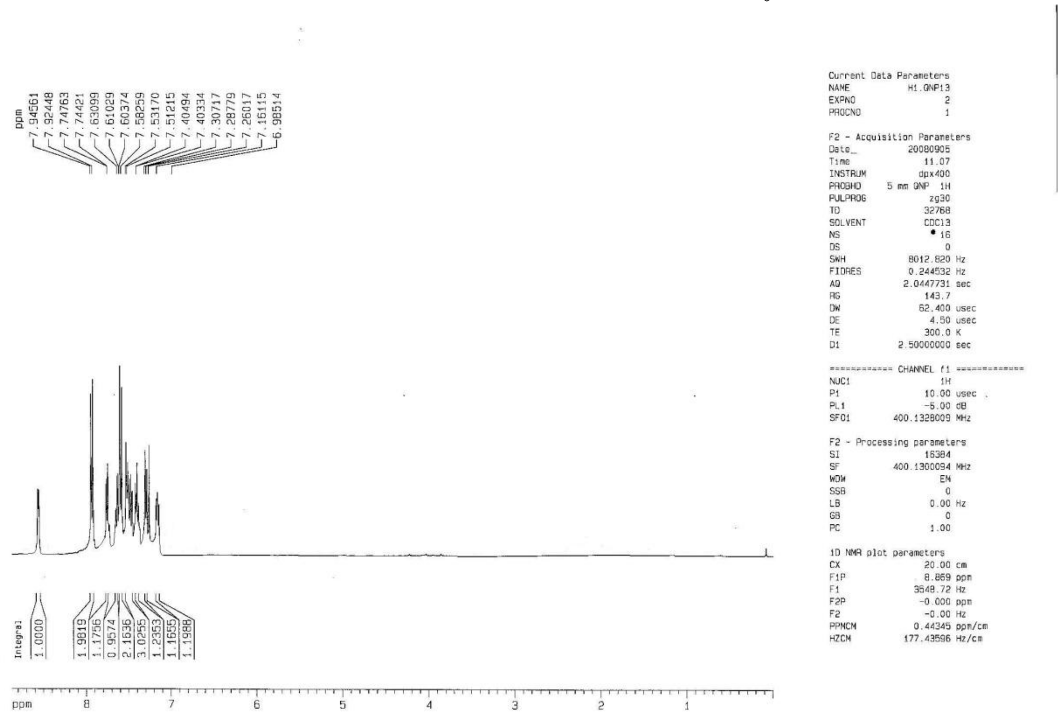

^{13}C-NMR of **2r**

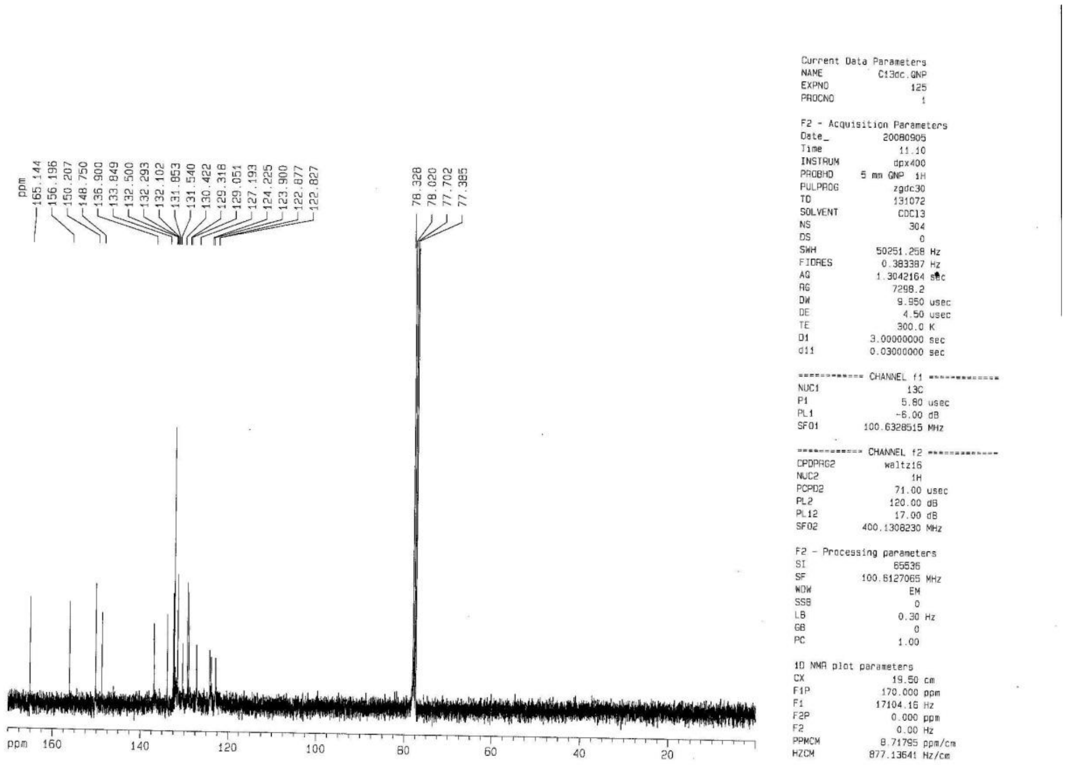

^{1}H-NMR of **2s**

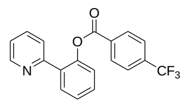

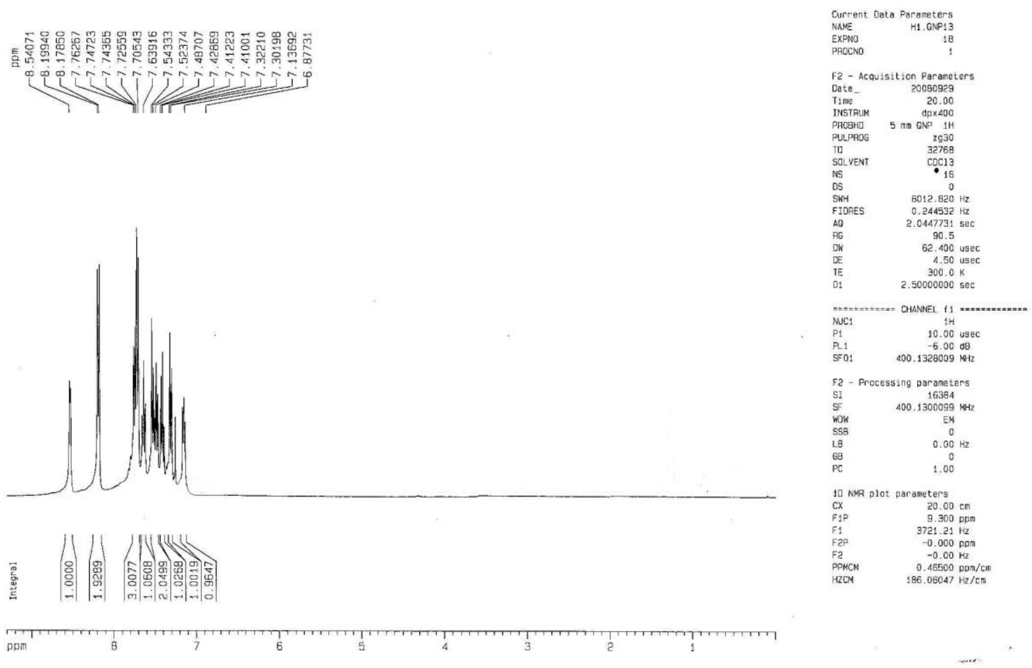

^{13}C-NMR of **2s**

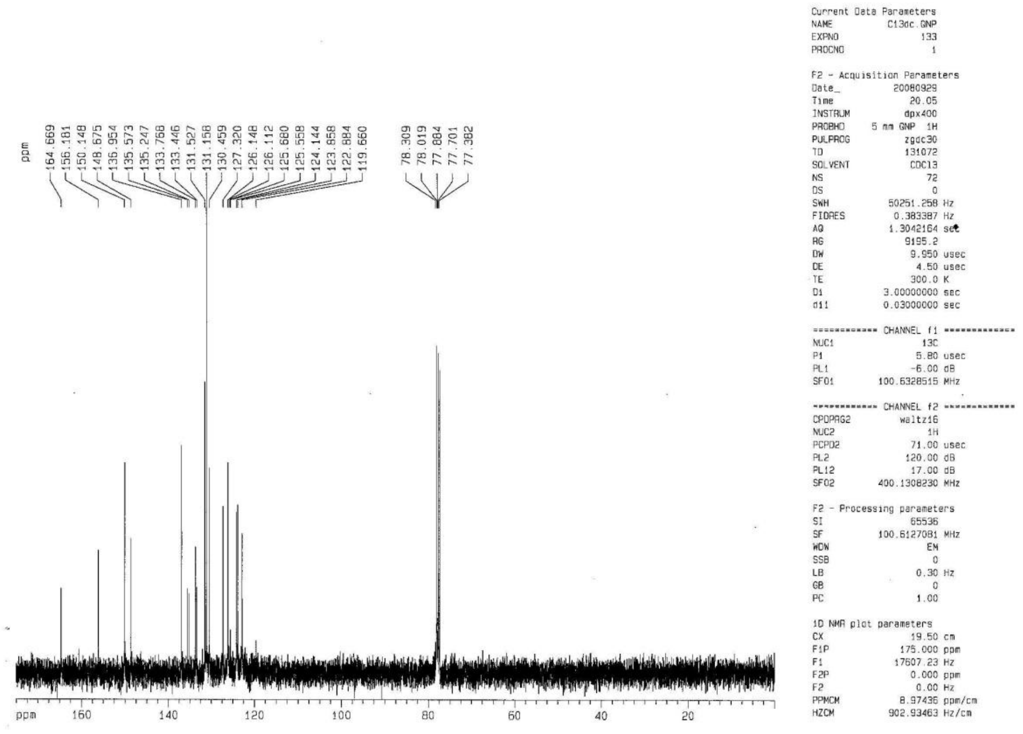

^{1}H-NMR of **2t**

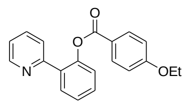

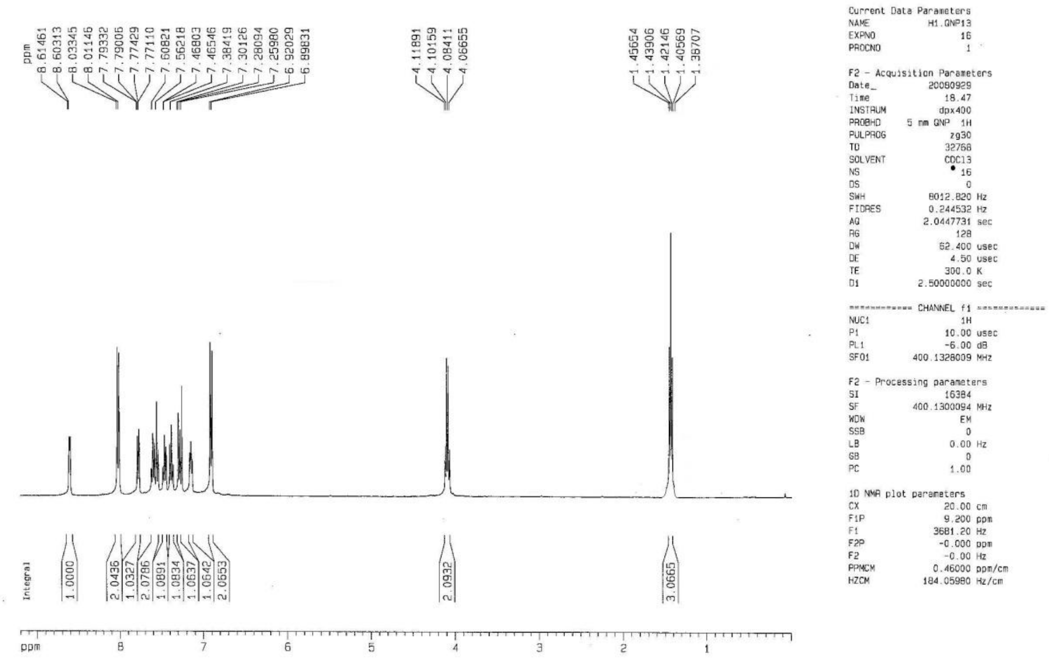

^{13}C-NMR of **2t**

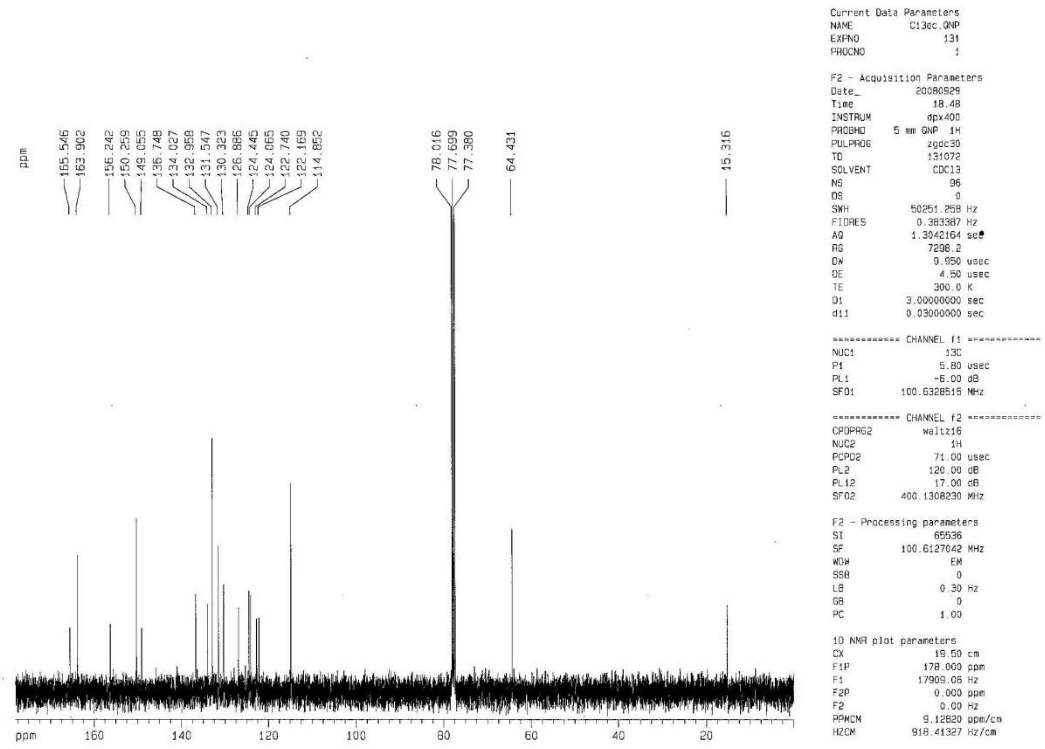

^{1}H-NMR of **2u**

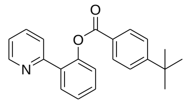

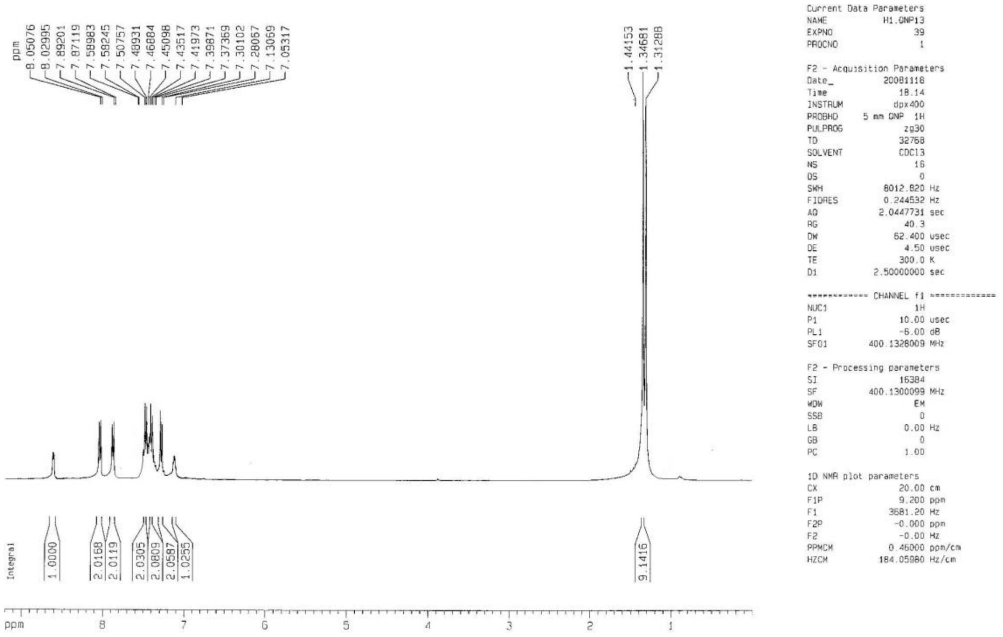

^{13}C-NMR of **2u**

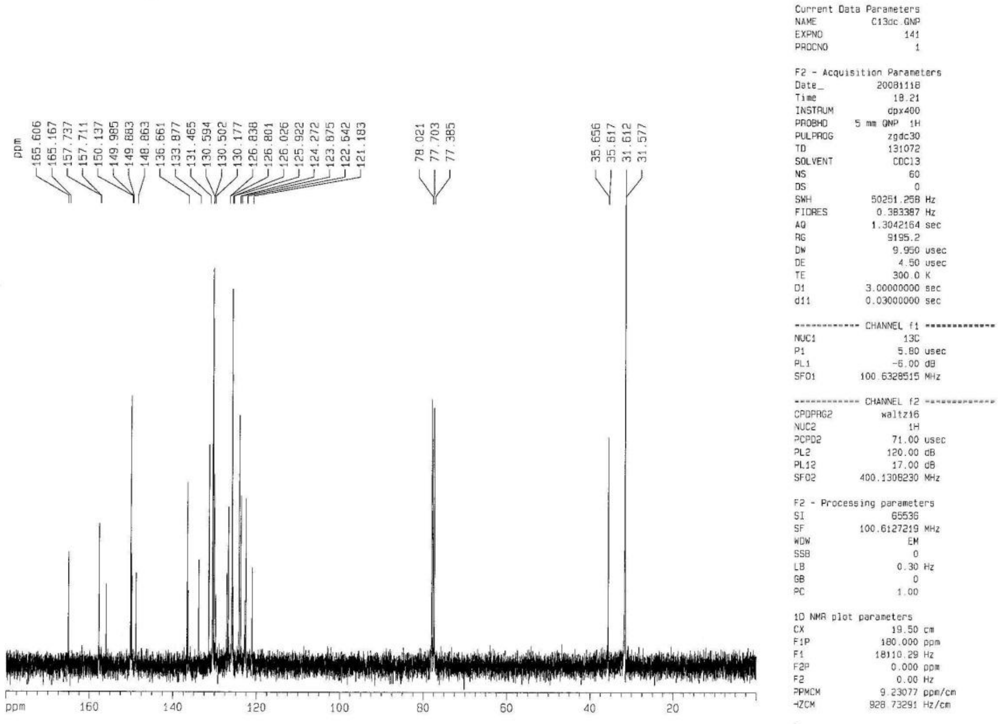

^{1}H-NMR of **2v**

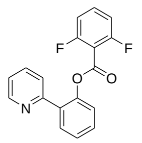

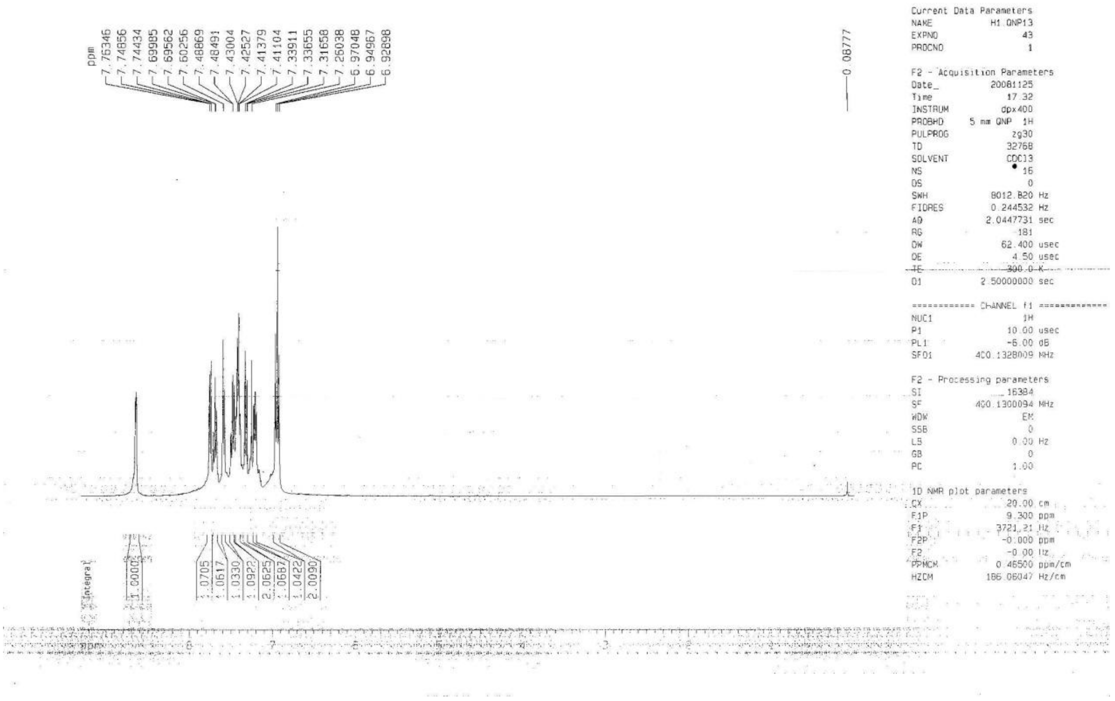

^{13}C-NMR of **2v**

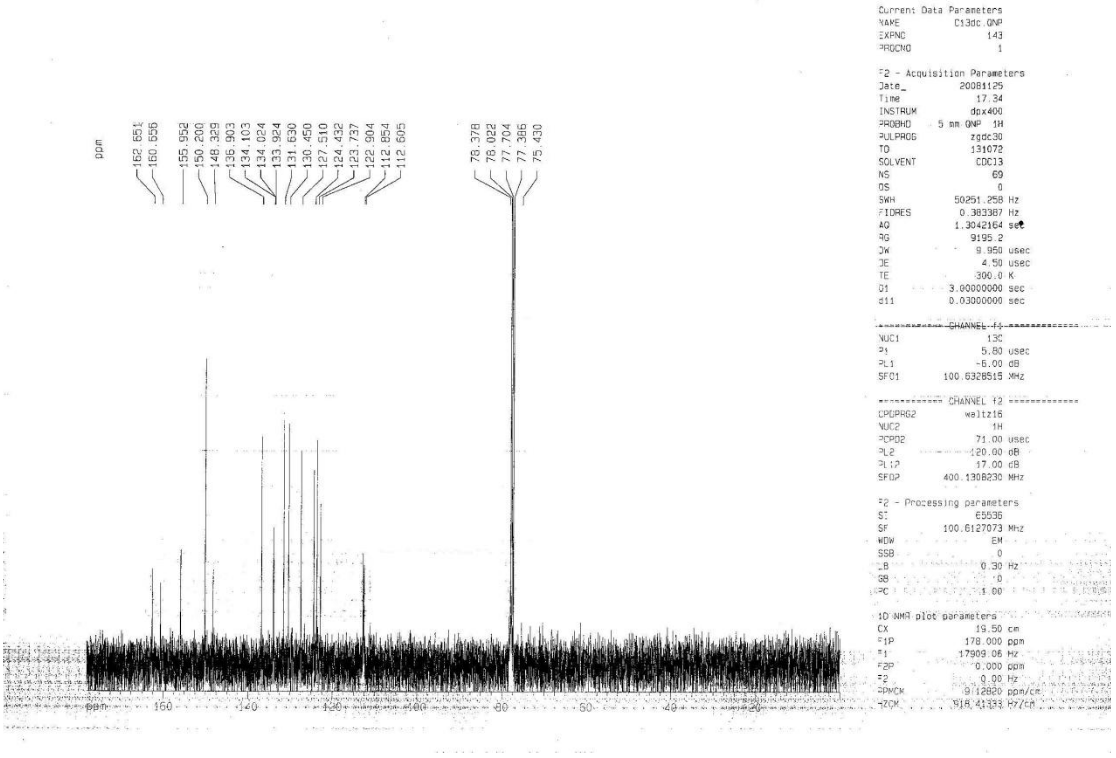

^{1}H-NMR of **2w**

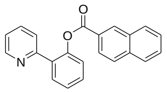

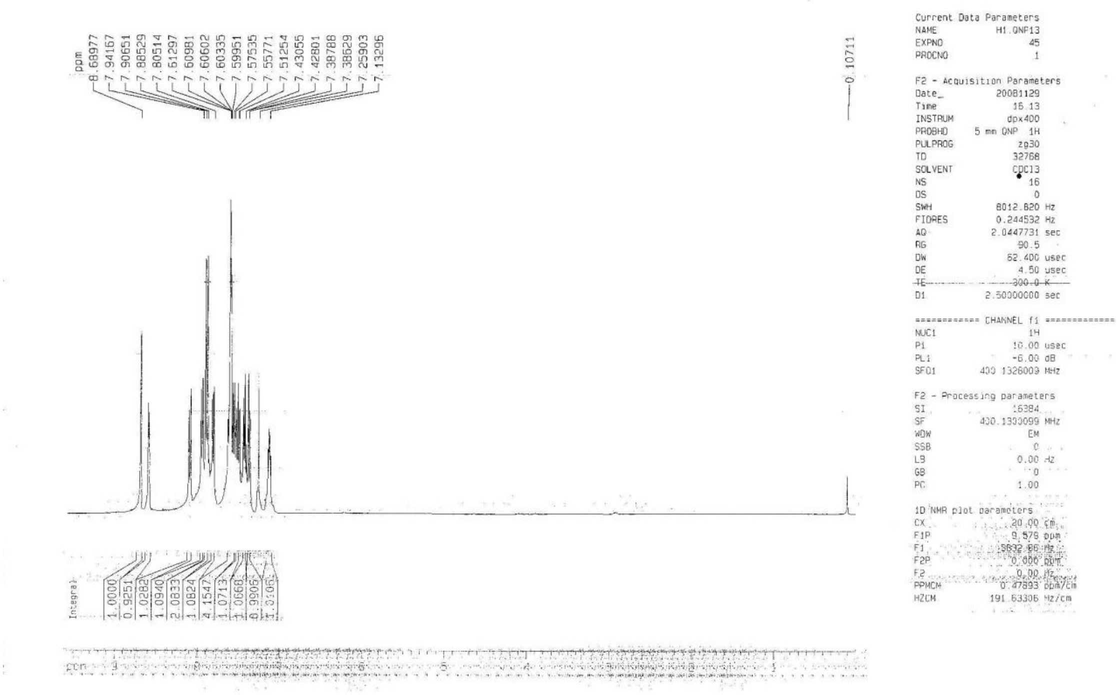

^{13}C-NMR of **2w**

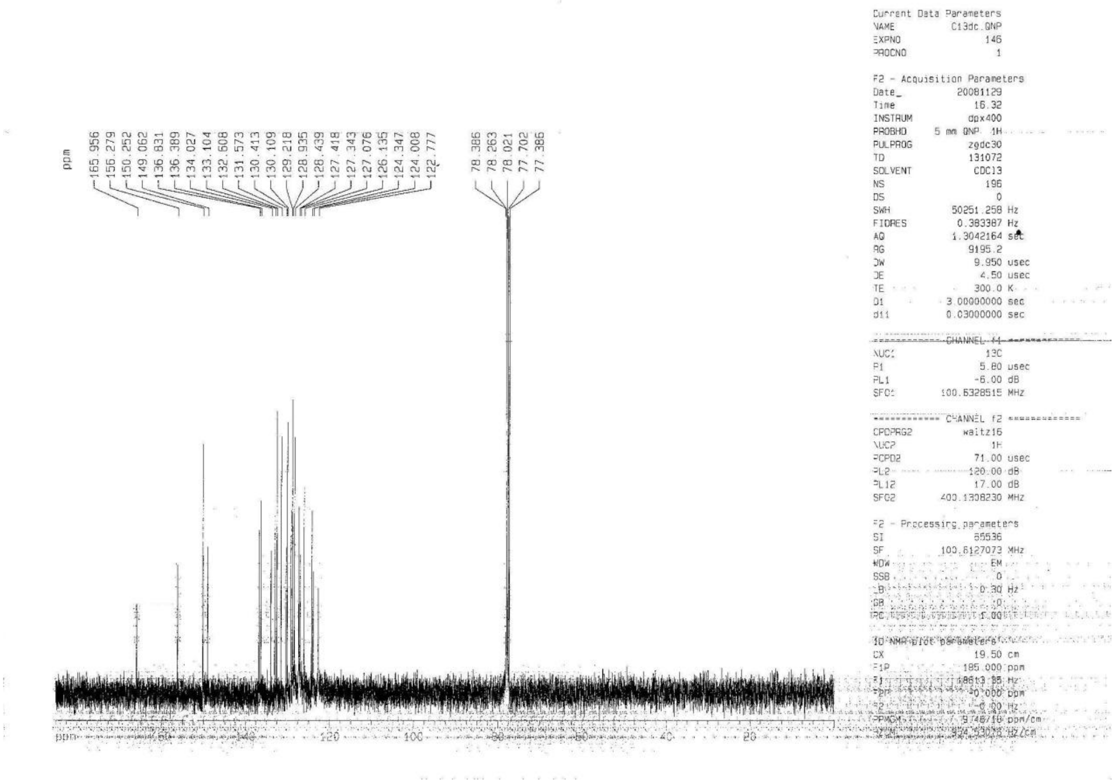

Supplement: Supplementary file 1 [file molecules-18-04403-s001.pdf]
